# Swarm Intelligence-Enhanced Detection of Non-Small-Cell Lung Cancer Using Tumor-Educated Platelets

**DOI:** 10.1016/j.ccell.2017.07.004

**Published:** 2017-08-14

**Authors:** Myron G. Best, Nik Sol, Sjors G.J.G. In ‘t Veld, Adrienne Vancura, Mirte Muller, Anna-Larissa N. Niemeijer, Aniko V. Fejes, Lee-Ann Tjon Kon Fat, Anna E. Huis In ‘t Veld, Cyra Leurs, Tessa Y. Le Large, Laura L. Meijer, Irsan E. Kooi, François Rustenburg, Pepijn Schellen, Heleen Verschueren, Edward Post, Laurine E. Wedekind, Jillian Bracht, Michelle Esenkbrink, Leon Wils, Francesca Favaro, Jilian D. Schoonhoven, Jihane Tannous, Hanne Meijers-Heijboer, Geert Kazemier, Elisa Giovannetti, Jaap C. Reijneveld, Sander Idema, Joep Killestein, Michal Heger, Saskia C. de Jager, Rolf T. Urbanus, Imo E. Hoefer, Gerard Pasterkamp, Christine Mannhalter, Jose Gomez-Arroyo, Harm-Jan Bogaard, David P. Noske, W. Peter Vandertop, Daan van den Broek, Bauke Ylstra, R. Jonas A. Nilsson, Pieter Wesseling, Niki Karachaliou, Rafael Rosell, Elizabeth Lee-Lewandrowski, Kent B. Lewandrowski, Bakhos A. Tannous, Adrianus J. de Langen, Egbert F. Smit, Michel M. van den Heuvel, Thomas Wurdinger

**Affiliations:** 1 Department of Neurosurgery, VU University Medical Center, Cancer Center Amsterdam, De Boelelaan 1117, 1081 HV Amsterdam, the Netherlands; 2 Department of Pathology, VU University Medical Center, Cancer Center Amsterdam, De Boelelaan 1117, 1081 HV Amsterdam, the Netherlands; 3 Brain Tumor Center Amsterdam, VU University Medical Center, Cancer Center Amsterdam, De Boelelaan 1117, 1081 HV Amsterdam, the Netherlands; 4 Department of Neurology, VU University Medical Center, Cancer Center Amsterdam, De Boelelaan 1117, 1081 HV Amsterdam, the Netherlands; 5 Department of Thoracic Oncology, The Netherlands Cancer Institute – Antoni van Leeuwenhoek Hospital, Plesmanlaan 121, 1066 CX Amsterdam, the Netherlands; 6 Department of Pulmonary Diseases, VU University Medical Center, De Boelelaan 1117, 1081 HV Amsterdam, the Netherlands; 7 Clinical Institute of Laboratory Medicine, Medical University of Vienna, Spitalgasse 23, 1090 Vienna, Austria; 8 Department of Radiation Sciences, Oncology, Umeå University, 90185 Umeå, Sweden; 9 MS Center Amsterdam, VU University Medical Center, De Boelelaan 1117, 1081 HV Amsterdam, the Netherlands; 10 Department of Surgery, VU University Medical Center, Cancer Center Amsterdam, De Boelelaan 1117, 1081 HV Amsterdam, the Netherlands; 11 Department of Clinical Genetics, VU University Medical Center, Cancer Center Amsterdam, De Boelelaan 1117, 1081 HV Amsterdam, the Netherlands; 12 thromboDx B.V., 1098 EA Amsterdam, the Netherlands; 13 Department of Neurology, Massachusetts General Hospital and Neuroscience Program, Harvard Medical School, 149 13^th^ Street, Charlestown, MA 02129, USA; 14 Department of Medical Oncology, VU University Medical Center, Cancer Center Amsterdam, De Boelelaan 1117, 1081 HV Amsterdam, the Netherlands; 15 Department of Surgery, Amsterdam Medical Center, Meibergdreef 9, 1105 AZ Amsterdam, the Netherlands; 16 Department of Experimental Cardiology, Utrecht University Medical Center, Heidelberglaan 100, 3584 CX Utrecht, the Netherlands; 17 Laboratory of Clinical Chemistry and Hematology, Utrecht University Medical Center, Heidelberglaan 100, 3584 CX Utrecht, the Netherlands; 18 Department of Clinical Chemistry, The Netherlands Cancer Institute – Antoni van Leeuwenhoek Hospital, Plesmanlaan 121, 1066 CX Amsterdam, the Netherlands; 19 Department of Pathology, Princess Máxima Center for Pediatric Oncology and University Medical Center Utrecht, Lundlaan 6, 3584 EA Utrecht, the Netherlands; 20 Translational Research Unit, Dr. Rosell Oncology Institute, Quirón Dexeus University Hospital, Calle Sabine Arana 5-19, 08028 Barcelona, Spain; 21 Pangaea Biotech SL, Calle Sabine Arana 5-19, 08028 Barcelona, Spain; 22 Catalan Institute of Oncology, Hospital Germans Trias i Pujol, Carretera de Canyet, 08916 Barcelona, Spain; 23 Molecular Oncology Research (MORe) Foundation, Calle Sabine Arana 5-19, 08028 Barcelona, Spain; 24 Department of Pathology, Massachusetts General Hospital, Harvard Medical School, 149 13^th^ Street, Charlestown, MA 02129, USA; 25 Department of Respiratory Diseases, Radboud University Medical Center, 6500 HB Nijmegen, the Netherlands

**Keywords:** tumor-educated platelets, blood platelets, RNA, cancer diagnostics, particle-swarm optimization, splicing, swarm intelligence, self-learning algorithms, liquid biopsies, NSCLC

## Abstract

Blood-based liquid biopsies, including tumor-educated blood platelets (TEPs), have emerged as promising biomarker sources for non-invasive detection of cancer. Here we demonstrate that particle-swarm optimization (PSO)-enhanced algorithms enable efficient selection of RNA biomarker panels from platelet RNA-sequencing libraries (n = 779). This resulted in accurate TEP-based detection of early- and late-stage non-small-cell lung cancer (n = 518 late-stage validation cohort, accuracy, 88%; AUC, 0.94; 95% CI, 0.92–0.96; p < 0.001; n = 106 early-stage validation cohort, accuracy, 81%; AUC, 0.89; 95% CI, 0.83–0.95; p < 0.001), independent of age of the individuals, smoking habits, whole-blood storage time, and various inflammatory conditions. PSO enabled selection of gene panels to diagnose cancer from TEPs, suggesting that swarm intelligence may also benefit the optimization of diagnostics readout of other liquid biopsy biosources.

## Highlights

• Tumor-educated platelet (TEP) RNA profiles allow for blood-based cancer diagnostics
• Inflammatory conditions only minimally confound TEP-based cancer detection
• Swarm intelligence algorithms enable efficient selection of biomarker gene panels
• TEP gene panels enable support vector machine-based classification of lung cancer

## Graphical Abstract

**Figure:**
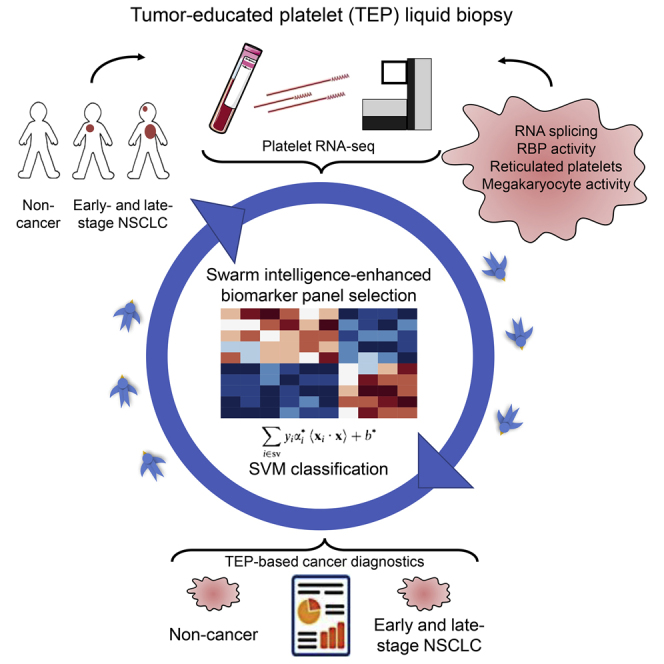

## Teaser

Best et al. use particle-swarm optimization algorithms and RNA-seq of tumor-educated platelets from patients to generate RNA sets capable of identifying patients with non-small-cell lung cancer, including those having early stage, from individuals without cancer, including those having inflammatory conditions.

## Significance

**Detection of cancer in a minimally invasive manner is considered the holy grail for cancer diagnostics. A notorious challenge is the identification of optimal biomarker panels from such liquid biosources. To select robust biomarker panels for disease classification the use of “swarm intelligence” was proposed, especially particle-swarm optimization (PSO). PSO-driven algorithms are inspired by the concomitant swarm of birds and schools of fish that by self-organization efficiently adapt to their environment. Here, PSO algorithms are exploited for the identification of optimal biomarker gene lists, resulting in a tumor-educated platelet RNA biomarker panel that discriminates patients with NSCLC from healthy individuals and patients with various non-cancerous inflammatory conditions. Follow-up analysis of additional early-stage cancer patients is warranted.**

## Introduction

Non-invasive collection of cancer-associated circulating biomarkers enables efficient, rapid, and detailed molecular characterization of tumors. Recent advancements in isolation and characterization of cell-free DNA, plasma RNA, circulating proteins, circulating tumor cells, extracellular vesicles, and tumor-educated platelet (TEP) RNA facilitated detection of cancer-specific genomic and transcriptomic aberrations in blood (Alix-Panabières and Pantel, 2016, Best et al., 2015, Bettegowda et al., 2014, Chan et al., 2013, Newman et al., 2016, Nilsson et al., 2011, Nilsson et al., 2015, Skog et al., 2008, Wan et al., 2017). Blood platelets act as local and systemic responders during tumorigenesis and cancer metastasis (McAllister and Weinberg, 2014), thereby being exposed to tumor-mediated platelet education, and resulting in altered platelet behavior (Kerr et al., 2013, Labelle et al., 2011, Schumacher et al., 2013). We have previously demonstrated that TEP RNA can function as a biomarker trove to detect and classify cancer from blood via self-learning support vector machine (SVM)-based algorithms (Best et al., 2015). We termed this highly multiplexed biomarker signature detection platform thromboSeq. In this study, we investigated the potential and origin of spliced RNA profiles from TEPs for the non-invasive detection of early- and late-stage non-small-cell lung cancer (NSCLC).

## Results

### Platelet Collection for the Detection of NSCLC

Blood platelets were collected of a cohort of NSCLC patients (n = 402; n = 57 early locally advanced and n = 344 metastasized late-stage [n = 1 unknown]) and individuals with no known cancer, but not excluding individuals with inflammatory diseases (n = 377), for analysis by thromboSeq (Figure S1A; Table S1). Importantly, extrinsic factors can be of influence in the selection process of the platelet RNA biomarker panels (Diamandis, 2016, Feller and Lewitzky, 2016, Joosse and Pantel, 2015). By statistical modeling of a previous thromboSeq dataset, which is publicly available (Best et al., 2015), we were able to confirm that the age of the individual and blood storage time can influence the platelet classification score (p value 0.002 and 0.09, respectively, Table 1). Although the contribution of blood storage is not statistically significant, we do not exclude that the observed trend could result in a statistically significant contribution in a larger dataset. Hence, we first assembled a subcohort of blood platelet samples from patients with NSCLC (n = 159; n = 6 early locally advanced, n = 153 metastasized late-stage) and individuals with no known cancer (n = 104), matched for age (median age, interquartile range [IQR] of 61 [14.5] and 58 [12.25] years, respectively), smoking status, and blood storage time (platelet isolation within 12 hr of blood collection) (Table 2). This matched NSCLC/non-cancer cohort allowed us to assess the contribution of potential technical and biological variables, and to investigate the platelet RNA profiles and RNA-processing pathways. Absence of platelet activation during blood collection and storage was confirmed by stable levels of the platelet activation-dependent surface markers P-selectin and CD63, as measured by flow cytometry (n = 6) and similarly as observed for the negative control, but in contrast to platelets artificially activated with 20 μM TRAP (Figure S1B).

**Table 1:** Comprehensive Overview of the Study Cohort and Statistical Contribution to the Classifiers

Cohort	Group	n	Acc. (%)	AUC (95% CI)	No. with Inflammatory Disease	Median Age (IQR)	Blood Storage (% <12 hr)	Statistical Predictive Contribution Likelihood Ratio Chi-Square Value (p Value)				
								Patient Age	Blood Storage	Gender	Smoking	thromboSeq Classification
**Unmatched Cohort (**Best et al., 2015**)**												

Training (unmatched)	healthy	39	92	0.99 (0.97–1.00)	0	40 (22.25)	100	9.8 (p = 0.002)	2.9 (p = 0.09)	0.8 (p = 0.38)	NA	29.5 (p < 0.0001)
	NSCLC	36			NA	59 (13.25)	61					
Validation (unmatched)	healthy	16	98	0.98 (0.93–1.00)	0	32.5 (26.25)	100	0.004 (p = 0.95)	0.01 (p = 0.90)	3.5 (p = 0.06)	NA	21.6 (p < 0.0001)
	NSCLC	24			NA	62 (14.25)	58					

**Matched Cohort (This Study) Genes: n = 830**												

Training (matched)	non-cancer	44	77	0.84 (0.75–0.92)	36	62 (18.5)	100	2.4 (p = 0.12)	NA	0.03 (p = 0.87)	5.7 (p = 0.12)	30.7 (p < 0.0001)
	NSCLC	49			NA	59 (9)	100					
Evaluation (matched)	non-cancer	20	85	0.91 (0.82–1.00)	4	61 (10.25)	100	4.1 (p = 0.04)	NA	0.05 (p = 0.80)	6.0 (p = 0.11)	32.0 (p < 0.0001)
	NSCLC	20			NA	58 (24)	100					
Validation (matched)	non-cancer	40	91	0.95 (0.91–0.99)	9	56 (9.25)	100	3.7 (p = 0.06)	NA	0.1 (p = 0.95)	14.7 (p = 0.002)	76.2 (p < 0.0001)
	NSCLC	90			NA	63 (14)	100					

**Full Cohort (This Study) Genes: n = 1,000**												

Training (matched)	non-cancer	60	84	0.90 (0.84–0.95)	30	59 (9.25)	100	<0.0001 (p = 0.99)	NA	3.4 (p = 0.18)	2.7 (p = 0.43)	58.7 (p < 0.0001)
	NSCLC	60			NA	61 (13.25)	100					
Evaluation (matched)	non-cancer	44	91	0.93 (0.87–0.99)	19	58 (15.5)	100	0.62 (p = 0.43)	NA	1.1 (p = 0.30)	9.9 (p = 0.02)	55.0 (p < 0.0001)
	NSCLC	44			NA	62 (13)	100					
Late-stage validation (unmatched)	non-cancer	273	88	0.94 (0.92–0.96)	94	40 (20)	97	39.6 (p < 0.0001)	0.07 (p = 0.80)	0.19 (p = 0.67)	33.5 (p < 0.0001)	91.5 (p < 0.0001)
	NSCLC	245			NA	64 (14)	75					
Loc.-adv. validation (unmatched)	non-cancer	53	81	0.89 (0.83–0.95)	8	53 (12)	98	23.4 (p < 0.0001)	4.5 (p = 0.03)	3.6 (p = 0.06)	25.6 (p < 0.0001)	26.7 (p < 0.0001)
	NSCLC	53			NA	62 (11)	83					

NA, not applicable. See also Table S1.

**Table 2:** Demographics of Patient Age, Smoking, and Blood Storage Time-Matched Cohort

Characteristics	non-cancer (n = 104)	NSCLC (n = 159)
Gender (male, %)	45 (43)	83 (53)
Median age (IQR, min-max)	58 (12.25, 46–86)	61 (14.5, 27–88)
Smoking (current, %)	13 (13)	32 (20)
Smoking (former, %)	16 (15)	39 (24)
Smoking (never, %)	65 (62)	66 (42)
Smoking (unknown, %)	10 (10)	22 (14)
Distant metastasis	NA	152 (unknown: 1)

IQR, interquartile range; NA, not applicable. See also Table S1.

### Technical Performance Parameters of thromboSeq

**Figure 1:**
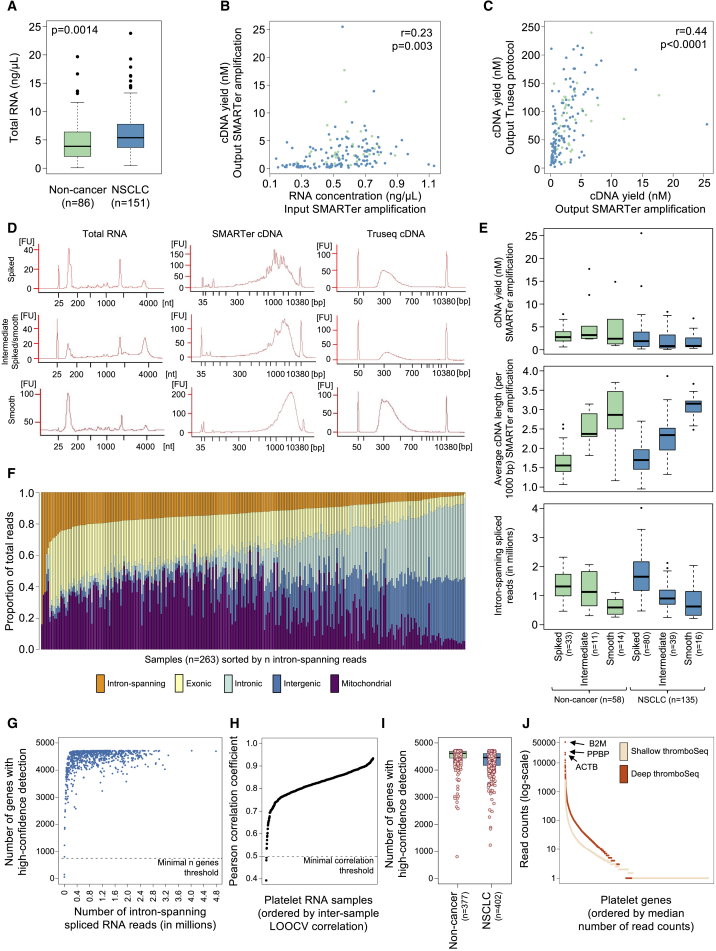
Technical Performance of thromboSeq (A) Platelet total RNA yield in ng/μL isolated from 6 mL whole blood in EDTA-coated Vacutainers tubes. p Value calculated by independent Student's t test. (B) Correlation plot (Pearson's correlation) of estimated RNA input to the output SMARTer cDNA yield. Each dot represents a sample, color-coded by clinical group. (C) Correlation plot (Pearson's correlation) of SMARTer cDNA yield to the Truseq cDNA library yield. Each dot represents a sample, color-coded by clinical group. (D) Bioanalyzer profiles of samples with spiked, smooth, and intermediate spiked/smooth traces for total RNA as measured by RNA 6000 Picochip (left column), SMARTer amplified cDNA as measured by DNA High Sensitivity chip (middle column), and Truseq cDNA libraries as measured by DNA 7500 chip (right column). (E) Shown are for spiked, smooth, and intermediate spiked/smooth SMARTer cDNA trace profiles for both non-cancer and NSCLC are the relative cDNA yield in nmol following SMARTer amplification (top), average cDNA length per 1,000 bp following SMARTer amplification (middle), and the number of intron-spanning spliced RNA reads (bottom). (F) Selection of intron-spanning spliced RNA reads for thromboSeq analysis. Stackplot indicates the distribution of reads for each sample, subspecified from intron-spanning (orange), exonic (yellow), intronic (green), intergenic (blue), and mitochondrial (purple) regions. (G) Selection of samples with >750 genes detected for thromboSeq analysis. (H) Leave-one-sample-out cross-correlation filtering step for which counts of each sample were correlated to the median counts of all other samples. (I) Number of genes detected with confidence in the platelet RNA samples using shallow thromboSeq. (J) Deep thromboSeq versus shallow thromboSeq for matched platelet samples. Median total read counts (min-max) in millions: 59.7 (43.2–96.2) for deep thromboSeq and 12.9 (11.6–20.0) for shallow thromboSeq. The three genes with highest expression in deep thromboSeq are highlighted. The boxes of the boxplots indicate the interquartile range (IQR), the horizontal black line indicates the median values, and the whiskers range 1.5× the IQR. See also Figure S1 and Table S1.

We isolated platelet samples from whole blood by a standardized differential centrifugation protocol and extracted total RNA. We previously observed only minor contamination of nucleated white blood cells (Best et al., 2015). We observed that 80% (111 out of 138) of the NSCLC patients assigned to the matched cohort, of which platelet counts were available at day of blood collection for thromboSeq, had platelet counts within the reference range (150–450 × 10^9^/L), and 16% of the NSCLC patients had thrombocytosis (>450 × 10^9^/L; Figure S1C). We evaluated the platelet RNA quality using the Bioanalyzer. We compared the total platelet RNA yield from 6 mL of whole blood of non-cancer individuals (n = 86) and NSCLC patients (n = 151) from the matched cohort. The average total RNA obtained from the blood samples is 146 ng (SD, 130 ng, n = 237 samples), and we observed a minor but significant increase in total RNA in platelets of NSCLC patients (median value non-cancer, 4.7 ng/μL [n = 86]; NSCLC, 6.5 ng/μL [n = 151], p = 0.0014, Student's t test; Figure 1A). The platelet RNA yield appeared to be moderately correlated to the platelet counts from a subset of the matched cohort (r = 0.24, p = 0.001, n = 171; Figure S1D). The increase in platelet RNA in NSCLC patients may be attributed to a potential difference in platelet turnover in NSCLC patients, resulting in more young RNA-rich platelets (Stone et al., 2012), although contribution of an increased quantity of platelets could not be excluded.

The platelet RNA samples were diluted to ∼500 pg/μL total RNA and subjected to SMARTer cDNA synthesis and amplification. Amplification of cDNA was confirmed by the Bioanalyzer, and followed by Truseq labeling. We observed a moderate correlation among total RNA input for SMARTer amplification and SMARTer cDNA yield (r = 0.23, p = 0.003, n = 177, Pearson's correlation; Figure 1B). SMARTer cDNA yield and Truseq cDNA yield correlated even stronger (r = 0.44, p < 0.0001, n = 167, Pearson's correlation; Figure 1C). The relatively moderate conversion efficiency of total RNA into amplified cDNA may be explained by the relatively high contribution of non-poly(A)-tailed RNAs, such as small non-coding RNAs, ribosomal RNAs, and circular RNAs, to the platelet RNA content (Alhasan et al., 2016, Bray et al., 2013, Landry et al., 2009). We confirmed that the platelet count and RNA yield did not correlate to the thromboSeq RNA input concentration (r = −0.01, p = 0.87, and r = −0.04, p = 0.57, respectively, n = 171, both Pearson's correlation), thus ensuring that the resulting RNA-sequencing (RNA-seq) signatures are independent of platelet counts (Figures S1E and S1F). We observed subtle differences in the SMARTer cDNA Bioanalyzer trace profiles (Figure 1D). The slopes of the SMARTer cDNA products could be subdivided in spiked, smooth, and intermediate spiked/smooth trace profiles, and do not tend to be patient specific (Figure 1E). Whereas the Bioanalyzer RNA profiles and Truseq cDNA profiles are similar among these three SMARTer groups (Figure 1D), the samples with a more smooth-like pattern resulted in a 38% reduction in total counts of intron-spanning spliced RNA reads, and a concomitant 6.2-fold increase in reads mapping to intergenic regions (Figures 1E and 1F). We measured the average length of concatenated reads mapped to intergenic regions for spiked and smooth samples separately using Bedtools, and observed that the majority of reads (>10.9% for spiked samples and >13.5% for smooth samples, n = 50 samples each) had an average fragment length (concatenated reads) of <250 nt, with a peak at 100–200 nt (Figure S1G). We attribute the differences in these cDNA profiles at least partly to “contaminating” plasma DNA retained during the platelet isolation procedure (Jiang and Lo, 2016).

To prevent potential “contaminating” DNA from contributing to our downstream computational platelet RNA analyses we selected only spliced intron-spanning RNA reads after sequencing. Raw RNA-seq data of platelets were subjected to a standardized RNA-seq alignment pipeline to determine the number of intron-spanning platelet RNA reads (Best et al., 2015). Of samples that yielded less than 0.2 × 10^6^ intron-spanning reads in total after sequencing, we again sequenced the original Truseq preparation of the sample and merged the read counts (in 52 samples). We excluded the genes that yielded <30 intron-spanning reads in >90% of the cohort for all platelet samples that were subjected to thromboSeq (n = 784). This resulted in a platelet RNA-seq library of 4,722 different spliced genes detected with sufficient coverage. For each sample, we quantified the number of genes for which at least one intron-spanning read was mapped, and excluded two samples with <750 detected spliced genes (Figure 1G). We performed a leave-one-sample-out cross-correlation analysis to exclude another three platelet samples that showed a low intersample correlation of <0.5 compared with all other samples (Figure 1H), resulting in the full cohort of 779 samples (Figures S1A and S1H). We observed in the platelet RNA a rich repertoire of spliced RNAs, including 4,000–5,000 different messenger and non-coding RNAs (Figure 1I). The spliced platelet RNA diversity is in agreement with previous observations of platelet RNA profiles (Best et al., 2015, Bray et al., 2013, Rowley et al., 2011).

We investigated if collection of more Single-Read 100 base pair (bp) RNA-seq reads (∼5× deeper, deep thromboSeq) of the platelet cDNA libraries (n = 12 healthy donors) could result in detection of more low-abundant spliced RNAs (Figure 1J). We selected from the deep thromboSeq dataset genes with the highest read count numbers, and filtered matching read counts from the shallow thromboSeq dataset accordingly. Increasing the average coverage of shallow thromboSeq ∼5× did not result in significantly enriched detection of low-abundant platelet genes. Hence, we continued with the “shallow” thromboSeq sequencing protocol.

### Analysis of the Spliced RNA Repertoire of TEPs from NSCLC Patients

**Figure 2:**
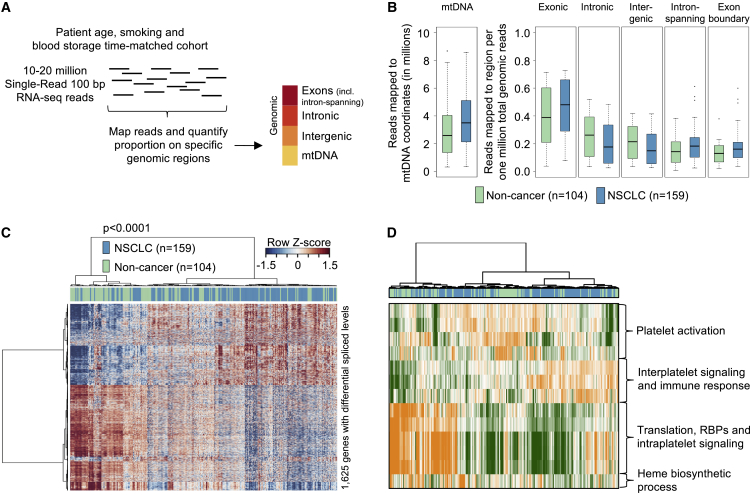
Analysis of the Spliced RNA Repertoire of TEPs from NSCLC Patients (A) Schematic figure represents the read distribution analyses procedure. A total of 100 bp reads were mapped to the human genome and reads mapping to four distinct regions were quantified. mtDNA, mitochondrial genome. (B) Boxplots indicate for non-cancer (green, n = 104) and NSCLC (blue, n = 159) the median and spread of reads mapping to mitochondrial (mtDNA), exonic, intronic, or intergenic regions, and the median and spread of intron-spanning and exon boundary reads (normalized to one million total genomic reads). The boxes of the boxplots indicate the IQR, the horizontal black line indicates the median values, and the whiskers range 1.5× the IQR. (C) Unsupervised hierarchical clustering of differentially spliced RNAs between non-cancer (n = 104) and NSCLC (n = 159) individuals, with FDR < 0.01. Columns indicate samples, rows indicate genes, and color intensity represents the *Z* score-transformed RNA expression values. Clustering of samples showed non-random partitioning (p < 0.0001, n = 263, Fisher's exact test). (D) PAGODA GO analysis. Most significant results by adjusted *Z* score, indicating high statistical significance, were clustered and visualized. Color code indicates a green-to-orange (low-to-high) score per sample per gene cluster. See also Table S2.

Distribution of the mapped platelet RNA-seq reads was investigated in samples assigned to the patient age, smoking, and blood storage time-matched NSCLC/non-cancer cohort (n = 263; Table 2). The mitochondrial genome and human genome, of which the latter includes exonic, intronic, and intergenic regions were quantified separately (Figures 2A and 2B). We observed an on average 1.2-fold increase in the number of reads mapping to the mitochondrial genome in NSCLC patients compared with cancer-free individuals (Figure 2B). In addition, we observed a 1.2-fold increase in the number of normalized reads (the reads per one million total genomic reads) mapping to exonic fractions in NSCLC patients, whereas for intronic and intergenic fractions the opposite was observed (Figure 2B). For samples with a larger proportion of reads mapping as intron-spanning spliced RNA reads, the contribution of reads mapping to the mitochondrial genome increased (r = 0.54, p < 0.0001, n = 263, Pearson's correlation), whereas the opposite was observed for reads mapping to intergenic regions (r = −0.54, p < 0.0001, n = 263, Pearson's correlation; Figures 1F and 2B). Despite the read distribution being partially confounded by Bioanalyzer cDNA trace profiles (data not shown), the prevalence of “smooth” and “spiked” samples is comparable among the matched non-cancer and NSCLC cohort (Figure 1E).

We selected the intron-spanning reads of 263 patient age, smoking, and blood storage time-matched individuals. Based on the intron-spanning read count analysis, we identified 1,625 spliced platelet genes with significantly differentially spliced levels (false discovery rate [FDR] < 0.01, 698 genes with enhanced splicing in platelets of NSCLC patients and 927 genes with decreased splicing in platelets of NSCLC patients) (Figure 2C; Table S2). Since we solely measured intron-spanning spliced sequences of a certain gene, we define enhanced or decreased splicing in genes in platelets as an increase or decrease of such reads compared with the control cohort, respectively. Of note, as platelets are devoid of a nucleus (Denis et al., 2005), the term differential expression does not seem appropriate for platelet RNA-seq analysis. The most significantly enriched spliced RNAs in TEPs included CFL1, ACOT7, and ARPC1B, whereas DDX5, RPS5, and EEF1B2 were decreased (Table S2). Unsupervised hierarchical clustering of intron-spanning reads separated the non-cancer and NSCLC samples into two distinct groups (p < 0.0001, Fisher's exact test; Figure 2C). These results indicate that a significant proportion of platelet RNA is differentially spliced in patients with NSCLC, independent of age, smoking status, and blood storage, as well as various inflammatory conditions.

PAGODA gene ontology (GO) analysis (Fan et al., 2016) was employed to functionally annotate the differentially spliced platelet RNAs in patients with NSCLC (Figure 2D). The most significant biological group (maximum adjusted *Z* score of 13.9) includes gene ontologies related to translation, RNA-binding proteins (RBPs), and intraplatelet signaling, with a low splicing score in NSCLC samples compared with non-cancer samples (Figure 2D). The most significantly enriched gene cluster in NSCLC patients compared with non-cancer individuals is related to interplatelet signaling and immune response (maximum adjusted *Z* score of 5.3). The clustering analysis identified correlations between platelet homeostasis in platelets of non-cancer individuals and specific immune signaling pathways in TEPs of NSCLC patients.

### Alternative Splicing and Exon Skipping in TEPs of Patients with NSCLC

**Figure 3:**
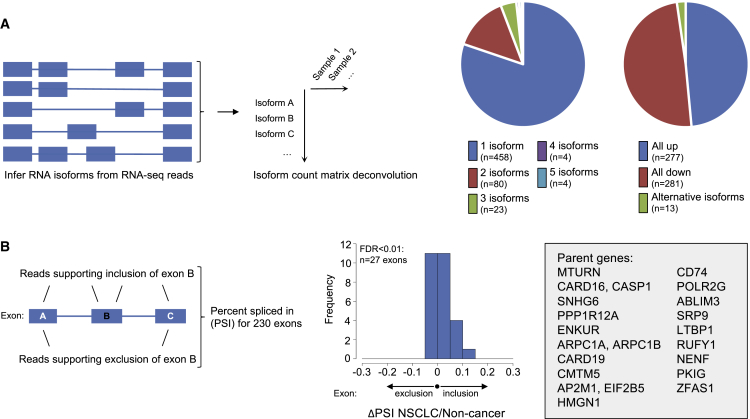
Alternative Splicing and Exon Skipping in TEPs of Patients with NSCLC (A) Schematic figure represents the development of an isoform count matrix that contains, per sample for each expressed RNA isoform, the number of reads supporting that particular isoform. RNA isoforms were inferred from the RNA-seq data using MISO. The isoform count matrix is employed for non-cancer versus NSCLC differential splicing analysis. The left pie chart indicates the gene loci (in total n = 571) with one or more differentially spliced isoforms (encoded in the color bars). Two gene loci with 10 isoforms each were not indicated. The right pie chart indicates the number of gene loci with multiple differentially spliced isoforms that show concordant up (blue box), concordant down (red box), or both directions (alternative isoforms; green box). (B) Schematic figure represents the MISO algorithm mapping exon-skipping events, thereby calculating the percent spliced in (PSI) value. Subsequently, ANOVA statistics were applied to individual exons with sufficient coverage in the dataset (n = 230) comparing read levels in non-cancer and NSCLC individuals, and assessed for overall in- or exclusion by calculation of the ΔPSI value. Histogram shows the direction of the PSI value for 27 exons with FDR < 0.01 (positive PSI values favors inclusion in NSCLC, whereas negative PSI values favors exclusion in NSCLC). Individuals gene names associated with the 27 exons are listed in the box.

For characterization of transcriptome-wide alternative isoforms and splicing events, we implemented the previously published MISO algorithm (Katz et al., 2010). We performed differential analysis between the RNA isoforms, and selected differential RNA isoforms between non-cancer individuals (n = 104) and NSCLC patients (n = 159) of the matched cohort (Figure 3A, left). Differential RNA isoform analysis revealed 743 RNA isoforms to be significantly enriched (n = 359) or depleted (n = 384) in TEPs of NSCLC patients. In 20% (113/571) of the genes, we identified multiple isoforms associated with the same gene locus (Figure 3A, left pie chart). However, in only 13/571 (2.3%) of the genes we observed potential alternative splicing of the isoforms (Figure 3A, right pie chart).

Next, we investigated alternative splicing events within genes, i.e., exon skipping. Here, we again applied the MISO algorithm to profile 38,327 annotated exons, and to infer the fraction of reads supporting either inclusion or exclusion of the particular exon compared with neighboring exons (schematic representation in Figure 3B). In addition, the MISO algorithm provides for each event a percent spliced in (PSI) value, quantifying the estimated fraction of reads supporting either inclusion or exclusion of a particular exon. For exon-skipping analysis, 230 exons remained eligible for analysis after filtering for exons with low coverage. By applying a threshold (ANOVA FDR < 0.01), we identified 27 (12%) exon-skipping events that were statistically significantly different in PSI value between non-cancer and NSCLC samples (n = 16 skipped in non-cancer, n = 11 skipped in NSCLC), and we observed a general trend toward exon inclusion in platelets of patients with NSCLC (Figure 3B). The putative exon-skipping events include the non-coding RNA SNHG6 (Chang et al., 2016), and the coding genes CD74 and SRP9 (Figure 3B). Previously, exon array analysis has revealed other exon inclusion events in NSCLC tumor tissue (Langer et al., 2010), possibly explaining this surrogate phenomenon and tendency toward exon inclusion in the TEPs of NSCLC patients.

### Correlation of Spliced Platelet RNA to P-Selectin Expression

**Figure 4:**
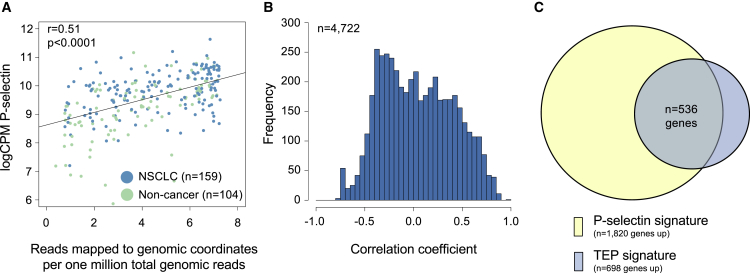
P-Selectin Signature (A) Correlation plot (Pearson's correlation) of proportion of reads mapping to exonic coordinates (x axis) versus the log_2_-transformed, RUV-corrected, and counts-per-million (logCPM) of P-selectin. Each dot represents a sample, colored by clinical group. (B) Distribution of Pearson's correlation coefficients of the correlation between logCPM levels of 4,722 genes and the logCPM of P-selectin. A subset of the genes show a strong positive correlation with P-selectin (r approximates 1), whereas others do not (r approximates 0). (C) Venn diagram overlay of genes upregulated in the NSCLC TEP signature and genes with a significant positive Pearson's correlation (FDR < 0.01) toward P-selectin. Number of overlapping genes is indicated in the Venn diagram.

The enrichment of total RNA yield from platelets of NSCLC patients (Figure 1A) suggests that these platelets contain more RNA molecules. Reticulated platelets were estimated to have an enriched RNA content of 20- to 40-fold (Angénieux et al., 2016, Hoffmann, 2014, Ingram and Coopersmith, 1969). Thus, we hypothesized that the platelet RNA of NSCLC patients is enriched with RNAs associated with younger platelets, including the membrane marker P-selectin (CD62), previously correlated to younger reticulated platelets (Bernlochner et al., 2016, Clancy et al., 2017). To determine the correlation between P-selectin levels and exonic read counts (surrogate for the unspliced RNA content in platelets), we compared the P-selectin counts-per-million values of 263 patient age, smoking, and blood storage time-matched individuals to the number of reads mapping to exons (r = 0.51, p < 0.0001, n = 263, Pearson's correlation; Figure 4A). We observed a moderate correlation between the platelet counts and the P-selectin levels (r = 0.19, p = 0.01, n = 171, Pearson's correlation; Figure S2), suggesting that thrombocytosis in patients with NSCLC is accompanied by an increase in younger reticulated platelets. We calculated Pearson's correlations of all individual genes (n = 4,722 in total) to the P-selectin expression levels, and compiled a P-selectin signature by selecting positively (r > 0) and most significantly (FDR < 0.01) correlated genes (n = 1,820 genes, Figure 4B). The P-selectin signature was enriched for markers such as CASP3, implicated in megakaryocyte-mediated pro-platelet formation (Morishima and Nakanishi, 2016), MMP1 and TIMP1, shown to be sorted into platelets (Cecchetti et al., 2011), and ACTB, previously detected in reticulated platelets (Angénieux et al., 2016, Clancy et al., 2017). Next, the P-selectin signature was compared with all differentially and increasingly spliced genes between non-cancer and NSCLC samples (Figure 4C). We observed that 77% (536/698) of genes in the P-selectin signature was also identified as significantly enriched in the TEPs of NSCLC patients (Figures 2C and 4C).

### Identification of RBP Signatures in TEP RNA Profiles

**Figure 5:**
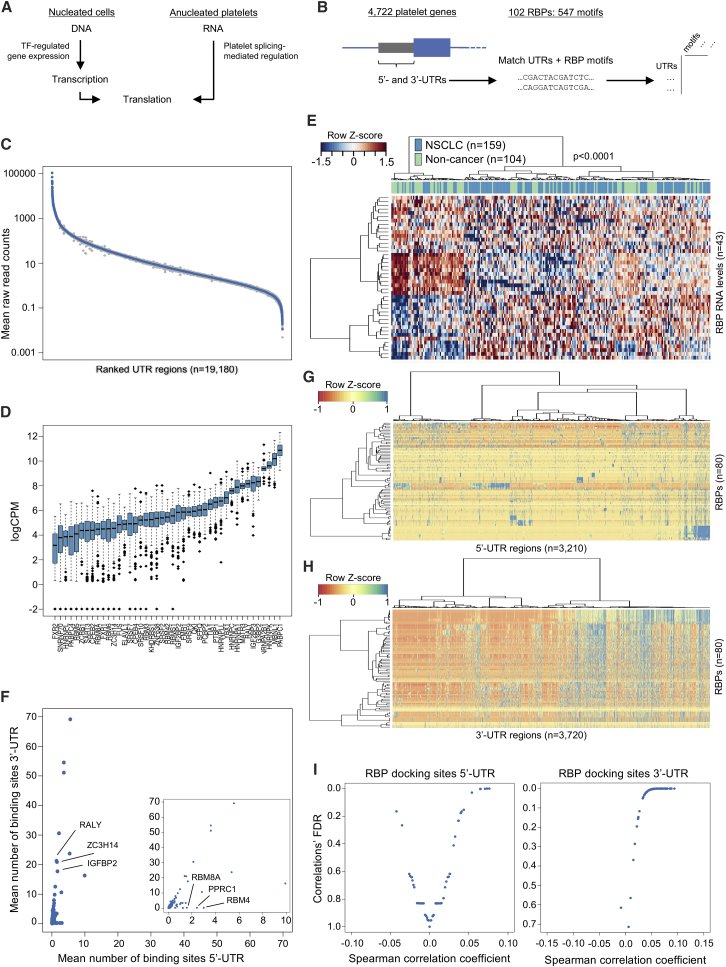
RNA-Binding Protein Analysis of TEP-Derived RNA Signatures (A) Schematic biological model highlighting the difference between nucleated cells and anucleated platelets in the context of regulation of translation. Nucleated cells (left) are able to regulate and maintain the transcriptome by transcription factor (TF)-mediated DNA transcription, in contrast to platelets. (B) Schematic representation of the RNA-binding protein (RBP)-thromboSearch engine algorithm. Reference genome sequences from 4,722 platelet genes were matched with 547 motif sequences deconvoluted from 102 RBPs. (C) UTR-read coverage filter. Blue dots represent mean counts across all samples, and gray shading indicates the respective standard deviations. (D) Boxplots indicate the average RBP RNA expression levels (n = 43 RBPs identified) in log_2_-transformed counts-per-million (logCPM) sorted by median normalized intron-spanning read level. The boxes of the boxplots indicate the IQR, the horizontal black line indicates the median values, and the whiskers range 1.5× the IQR. (E) Heatmap with unsupervised clustering of RNA levels from 43 RBPs in the age, smoking status, and blood storage time-matched cohort (n = 263 samples; n = 104 non-cancer, n = 159 NSCLC). The RBP RNA levels enable non-random clustering of the samples (p < 0.0001, n = 263, Fisher's exact test). (F) Enrichment of identified RBP binding sites per UTR region. The x and y axes represent the mean binding sites for the 5′ and 3′ UTR per RBP (dots, n = 80). Several RBPs are specifically enriched in the 3′ UTR, whereas others are enriched in the 5′ UTR. (G and H) Heatmap of all RBPs (rows) and all 5′ UTR (G) and 3′ UTR (H) regions detected with sufficient coverage in platelets (column). Number of binding sites is indicated in the color coding. (I) Spearman's rank correlation analysis between n binding sites of an RBP and the logarithmic fold-change (logFC) of genes in the NSCLC/non-cancer differential splicing analysis. Positive correlations indicate an enrichment in binding sites with an increase of the logFC, whereas negative correlations indicate the opposite. Plots indicate the relation between the Spearman's correlation coefficient (x axis) and the concomitant differential splicing FDR value.

Platelets contain a functional spliceosome and several splice factor proteins (Denis et al., 2005), and are able to splice pre-mRNA upon environmental queues (Denis et al., 2005, Rondina et al., 2011, Schwertz et al., 2006), resulting in protein translation (Weyrich et al., 1998). The inability of platelets to transcribe chromosomal DNA, as opposed to nucleated cells, prevents the platelets from transcription factor-mediated gene regulation, hinting at post-transcriptional regulation of the RNA pool, possibly by RBPs (Zimmerman and Weyrich, 2008). Indeed, the SF2/ASF- (SRSF1-) RBP has been implicated in splicing initiation of tissue factor mRNA in the platelets of healthy individuals (Schwertz et al., 2006). In general, RBPs are implicated in multiple co- and post-transcriptional processes associated with gene expression, such as RNA splicing, poly-adenylation, stabilization, and localization (Glisovic et al., 2008). The 5′ and 3′ UTR are considered to be the most prominent regulatory regions for pre-mRNAs (Ray et al., 2013), whereas intronic regions primarily mediate alternative splicing events such as exon skipping. SAGE analyses of platelet RNA lysates have shown that the platelets contain genes with an on average longer 3′ UTR length (Dittrich et al., 2006). Since our PAGODA GO analysis revealed RBP function as a key biological process affected in platelets of NSCLC patients (Figure 2D), we hypothesized that differential binding of RBPs to the UTR regions of platelet RNAs may at least partly explain the differential splicing patterns observed in TEPs (Figure 5A).

We developed an RBP-thromboSearch algorithm that scans for RBP binding motifs in UTR regions, and which identifies correlations between the number of binding sites and the log fold-change (logFC) of the particular gene (Figure 5B). We included 102 RBPs of which the binding motifs were previously identified (Ray et al., 2013). We only included UTR regions with sufficient read coverage in the RNA-seq data (Figure 5C), and identified 43 out of 102 RBPs with sufficient read coverage in the RNA profiles of the patient age, smoking, and blood storage time-matched dataset (Figure 5D). Visualization and clustering of these 43 RBPs resulted in non-random segregation between non-cancer (n = 104) and NSCLC (n = 159) (p < 0.001, Fisher's exact test), confirming the results of the PAGODA GO analyses, and possibly indicating that platelet mRNAs encoding for RBPs are differentially spliced in the presence of cancer (Figure 5E). We first identified RBPs with enriched tropism for either the 5′ or 3′ UTR, and observed that RBM4, PPRC1, and RBM8A were primarily targeted toward the 5′ UTR, whereas IGF2BP2, ZC3H14, and RALY showed an enriched binding repertoire for the 3′ UTR (Figure 5F). These enrichments were reported previously (Ray et al., 2013), supporting the specificity of our matching approach. All UTRs had at least one binding site for one of the RBPs. By analysis of the 3,210 5′ UTR regions and 3,720 3′ UTR regions, we observed that the number of RBP binding sites per UTR region showed a bimodal distribution, indicating controlled regulation of specific RBPs for specific UTR regions (Figures 5G and 5H). To assess whether the RNAs in the NSCLC TEP RNA signature are co-regulated by specific RBP binding sites, we correlated the logFC values of either the 5′ or 3′ UTR of the genes to the number of matching binding sides on either of these regions for each RBP. This resulted in five significant correlations for the 5′ UTR (FDR < 0.01, RBM4, RBM8A, PPRC1, FUS, and SAMD4A) and 69 for the 3′ UTR (FDR < 0.01, top five are PCBP1/2, SRSF1, RBM28 LIN28A, and CPEB2; Figure 5I).

### Particle-Swarm Optimization-Enhanced thromboSeq for NSCLC Diagnostics

**Figure 6:**
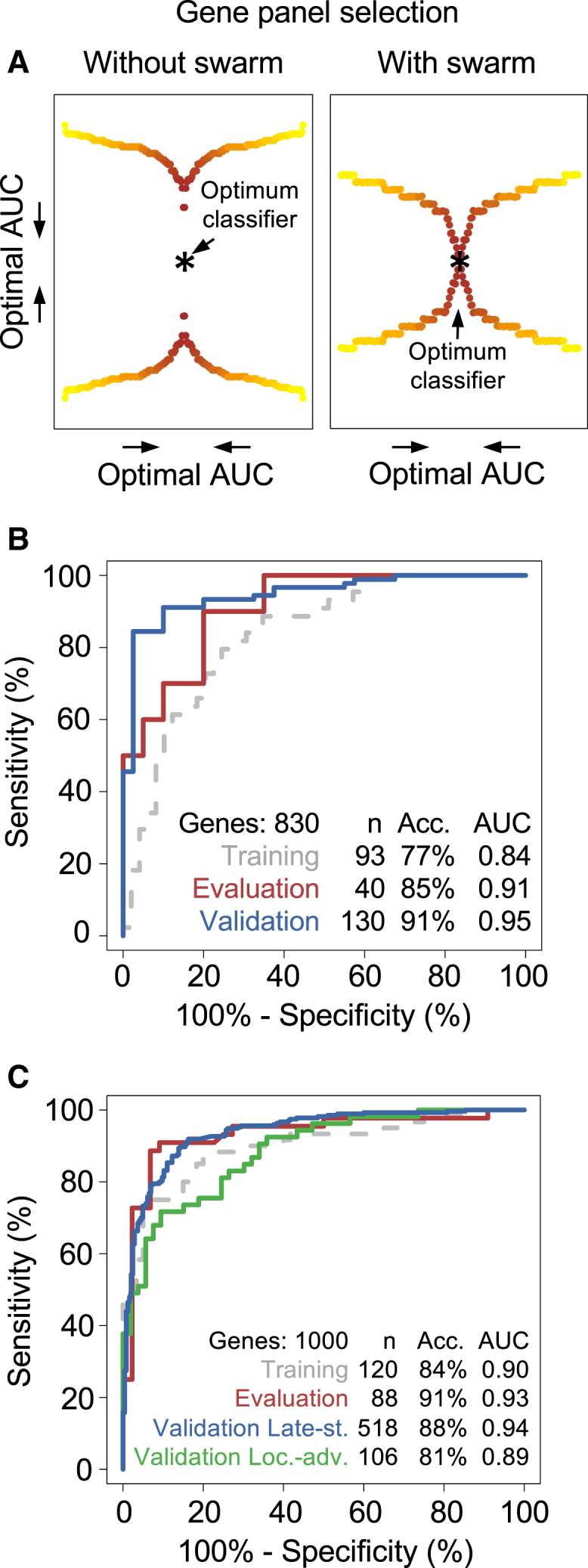
PSO-Enhanced thromboSeq for NSCLC Diagnostics (A) Schematic representation of the particle-swarm optimization (PSO) approach. Yellow-to-red colored dots represent AUC values of a cohort classified using a thromboSeq classification algorithm, with use of 100 randomly compiled biomarker gene panels (left) or 100 biomarker gene panels proposed by swarm intelligence (right). Dots were mirrored twice for visualization purposes. Most optimal AUC value reached by PSO-enhanced thromboSeq is indicated in both plots with an asterisk. (B) ROC analysis of PSO-enhanced thromboSeq classifications using only the matched non-cancer and NSCLC cohorts. Gray dashed line indicates ROC evaluation of the training cohort assessed by LOOCV, red line indicates ROC evaluation of the evaluation cohort (n = 40), blue line indicates ROC evaluation of the validation cohort (n = 130). Indicated are cohort size, most optimal accuracy, and AUC value. (C) ROC analysis of PSO-enhanced thromboSeq classifications using matched training and evaluation of non-cancer and NSCLC cohorts and validation in the remaining matched and unmatched samples. Gray dashed line indicates ROC evaluation of the training cohort assessed by LOOCV, red line indicates ROC evaluation of the evaluation cohort (n = 88). ROC evaluation of the late-stage NSCLC (Validation Late-st.; n = 518) and locally advanced NSCLC (Validation Loc.-adv.; n = 106) is plotted in the blue and green lines, respectively. Indicated are cohort size, most optimal accuracy, and AUC value. Acc., accuracy. See also Figures S3 and S4.

To develop an NSCLC diagnostics classification algorithm based on the differentially spliced platelet RNAs (Table S2), we employed the matched NSCLC/non-cancer platelet cohort (Table 2). We first improved the robustness of the data normalization procedure of our previously developed SVM-based thromboSeq classification algorithm (Best et al., 2015), by introduction of a remove unwanted variation (RUV)-based (Risso et al., 2014) iterative correction module, thereby considerably reducing the relative intersample variability (p < 0.0001, n = 263, Student's t test; Figures S3A–S3D). Second, we implemented a particle-swarm optimization (PSO)-driven meta-algorithm for selection of the most contributive genes used for classification (Figure S4A). The PSO-driven algorithm leverages the use of many candidate solutions (i.e., particles), and by adopting swarm intelligence and particle velocity the algorithm continuously searches for more optimal solutions, ultimately reaching the most optimal fit (Figure 6A) (Bonyadi and Michalewicz, 2017, Kennedy et al., 2001). Here, we favored PSO over other optimization algorithms because of the ease of implementation, fast convergence to acceptable solutions, the wide experience in the machine-learning field with this optimization algorithm, and the multiple PSO subvariants that have been developed (Bonyadi and Michalewicz, 2017). Finally, for testing the PSO-driven thromboSeq algorithm, we employed a separate training and evaluation cohort selected from the patient age, smoking, and blood storage time-matched dataset (n = 263 in total), and validated this in an independent matched validation cohort (Figure S4B). We summarized the predictive measures of the PSO-enhanced thromboSeq platform in a receiver operating characteristics (ROC) curve. We observed that this NSCLC classification algorithm has significant predictive power in evaluation (accuracy, 85%; area under the curve [AUC], 0.91; 95% confidence interval [CI], 0.82–1.00; n = 40; red line, Figure 6B) and independent validation cohorts (accuracy, 91%; AUC, 0.95; 95% CI, 0.91–0.99; n = 130; blue line, Figure 6B). *Post hoc* leave-one-out cross-validation (LOOCV) analysis of the training cohort suggests reduced performance (accuracy, 77%; AUC, 0.84; 95% CI, 0.75–0.92; n = 93; dashed gray line, Figure 6B), compared with the “matched” evaluation (85% accuracy) and validation cohort (91% accuracy). This may be explained by the different classification techniques used, and optimization of the gene panel toward the evaluation cohort at cost of classification power in the training cohort. Following PSO-enhanced gene panel selection, the performance metrics of the training, evaluation, and independent validation cohorts suggests that the algorithm has not been overfitted, a common pitfall of machine-learning tasks (Lever et al., 2016). The contribution of patient age, smoking status, and blood storage time to the cancer classification was negligible compared with the predictive power attributed to platelet RNA (Table 1). Of note, random selection and algorithm training using 1,000 other unique training samples sets from the matched cohort (n = 93 each), while locking the gene panel and validation cohort, showed similar classification strength (median AUC “validation cohort”, 0.85; IQR, 0.05), as opposed to random classification (median AUC “validation cohort”, 0.55; IQR, 0.01; p < 0.001).

Subsequently, we included all samples of the full non-matched NSCLC/non-cancer cohort (n = 402/n = 377, respectively) and developed a new classification algorithm. For development of the algorithm training cohort, we summed all matched patient age, smoking, and blood storage time-matched samples and assigned 120 samples for swarm-guided gene panel selection and SVM training, and 88 samples for swarm-based optimization. Again the training cohort of the NSCLC diagnostics classifier was not confounded by patient age, smoking status, or blood storage time (Table 1). A total of 571 samples (patient age, smoking, and/or blood storage time-unmatched), collected in multiple hospitals and from different clinical cohorts (Table S1), remained for validation of the algorithm. This cohort was divided into early-stage locally advanced NSCLC (n = 106; n = 53 NSCLC and n = 53 age-matched non-cancer) and late-stage metastatic NSCLC (n = 518; n = 245 NSCLC and n = 273 non-cancer) validation cohorts. These samples were predicted by the algorithm, while the algorithms' classification parameters were locked after training. We again summarized the predictive measures of the PSO-enhanced thromboSeq platform in ROC format, for evaluation (accuracy, 91%; AUC, 0.93; 95% CI, 0.87–0.99; n = 88; red line, Figure 6C), independent late-stage NSCLC validation (accuracy, 88%; AUC, 0.94; 95% CI, 0.92–0.96; p < 0.001; n = 518, blue line, Figure 6C), and independent (age-matched) locally advanced NSCLC validation cohorts (accuracy, 81%; AUC, 0.89; 95% CI, 0.83–0.95; p < 0.001; n = 106; green line, Figure 6C). *Post hoc* LOOCV analysis of the training cohort again resulted in reduced performance (accuracy, 84%; AUC, 0.90; 95% CI, 0.84–0.95; n = 120; dashed gray line, Figure 6C), compared with the “full” evaluation (91% accuracy), late-stage validation cohort (88% accuracy), and early-stage validation cohort (81% accuracy). Random selection of other training cohorts (n = 120 each), while locking the gene panel, resulted in similar classification strength (n = 1000, median AUC “validation cohort”, 0.89; IQR, 0.05), whereas for random classification the algorithm performance diminished (median AUC “validation cohort”, 0.67; IQR, 0.08; p < 0.001). From 49 NSCLC patients, two or more follow-up samples collected weeks to months following the first sample were included into the evaluation and/or independent validation cohorts. The difference between the lowest and highest classification score for a particular individual was 0.23 (SD, 0.15; n = 49 patients with a total of n = 123 samples). This suggests that the TEP educational program might result in slightly different platelet RNA profiles over time, possibly related to tumor growth or immune response.

## Discussion

Platelets actively maintain their RNA content while in circulation, including via the use of platelet RNA splicing (Denis et al., 2005, Rondina et al., 2011, Schwertz et al., 2006), circularization (Alhasan et al., 2016), and decay (Mills et al., 2016, Mills et al., 2017), possibly in response to external queues. Interestingly, we observed an enrichment of gene ontologies related to platelet activation in platelets of non-cancer individuals, whereas the isolated platelet pellets show no activation, as measured by classical activation analysis. The TEPs can perhaps behave in a “semi-activational state,” thereby also enhancing the risk for platelet-related events such as thrombosis. Altogether, our data indicate that the diagnostic TEP RNA profiles may be caused by (1) altered megakaryocytic RNA expression, (2) enrichment of reticulated platelets in patients with NSCLC (Dymicka-Piekarska and Kemona, 2008, Stone et al., 2012), (3) induction of splicing, possibly partially mediated by RBP activity and upstream regulatory kinases such as Clk (Denis et al., 2005, Schwertz et al., 2006), (4) sequestration of RNAs (Nilsson et al., 2011, Nilsson et al., 2015), and (5) alternative splicing events. Follow-up studies should address the biological mechanisms responsible for the TEP RNA signatures. This can possibly be achieved *in vitro* by “educating” cultured platelets with cancer cells or cancer cell-conditioned media, patient-derived platelet-depleted plasma, or studying platelet behavior and TEP RNA profiles collected from tumor xenograft mouse models. Also, the dynamic re-organization of the TEP signatures during therapy courses and disease progression should be investigated. Finally, follow-up studies should also investigate the contribution of RNA regulatory proteins on platelet RNA decay (Mills et al., 2016), intraplatelet RNA routing, and alternative splicing. Deep-splicing analysis by long-read RNA sequencing (Abdel-Ghany et al., 2016) might reveal distributions among spliced and unspliced TEP RNAs, and possibly uncover megakaryocyte-derived RNA expression programs. Since platelets originate from megakaryocytes in the bone marrow and the lung parenchyma (Lefrançais et al., 2017), these results are suggestive of tumor-bone marrow/lung parenchyma crosstalk in patients with NSCLC.

RNA-seq gene expression characterization, exploited by thromboSeq, provides a thorough, unbiased overview on the platelet RNA content. The strength of this platform is that, for any thromboSeq-based diagnostics test, the same RNA-seq wet-lab protocol can be applied resulting in unique spliced RNA profiles. The large gene panel selection is not a limitation for the application-specific software. Thus, targeted sequencing approaches may actually even limit the potential broad applicability of the platform. The algorithms use the RNA-seq input data to directly classify individuals based on bioinformatically selected gene panels specific for each diagnosis, an approach that is previously exploited for several tissue- or liquid biopsy-based classification algorithms (Moran et al., 2016, Newman et al., 2016, Veer et al., 2002). We conclude that the PSO-driven thromboSeq platform (Figure S4C) allows for robust biomarker selection for blood-based cancer diagnostics, independent of bias introduced by age of the individual, smoking habits, blood storage time, and certain inflammatory diseases. A further increase in the classification power of PSO-enhanced thromboSeq may be achieved by (1) training of the PSO-enhanced self-learning algorithms on significantly more matched sample cohorts, especially in the case of early-stage NSCLC samples, (2) evaluation of the swarm intelligence approach employing extended swarm intelligence algorithms such as the binary quantum-behaved PSO or genetic bee colony algorithms (Alshamlan et al., 2015, Xi et al., 2016), (3) including analysis of platelet-derived small RNAs (e.g., miRNAs), (4) including platelet-derived non-human RNAs, and/or (5) combining multiple blood-based biosources, such as TEP RNA, exosomal RNA, cell-free RNA, and cell-free DNA. The PSO-driven thromboSeq algorithm might also be applicable to other biosources and indications. At present, large scale validation of TEPs for the (early) detection of NSCLC and other tumor types is warranted.

## STAR★Methods

### Key Resources Table

**Table:** 

REAGENT or RESOURCE	SOURCE	IDENTIFIER
**Antibodies**		

APC anti-human CD41 antibody	Biolegend	Cat# 303710; RRID: AB_2249385
PE anti-human CD62P antibody	Biolegend	Cat# 304906; RRID: AB_314478
FITC anti-human CD63 antibody	Biolegend	Cat# 353005; RRID: AB_10933264

**Biological Samples**		

779 blood platelet samples	This study	Table S1

**Chemicals, Peptides, and Recombinant Proteins**		

OptiPrep Density Gradient Medium	Sigma-Aldrich	Cat# D1556
Multiplate TRAPtest	Roche	Cat# 06675883190
RNALater solution	Thermo Scientific	Cat# AM7020

**Critical Commercial Assays**		

mirVana miRNA isolation kit	Ambion, Thermo Scientific	Cat# AM1560
SMARTer Ultra Low RNA Kit for Illumina Sequencing version 3	Clontech	Cat# 634853
Truseq Nano DNA Sample Prep Kit	Illumina	Cat# FC-121-4001
RNA picochip and reagents, Bioanalyzer 2100	Agilent	Cat# 5067-1513
DNA 7500 chip and reagents, Bioanalyzer 2100	Agilent	Cat# 5067-1506
DNA High Sensitivity chip and reagents, Bioanalyzer 2100	Agilent	Cat# 5067-4626

**Deposited Data**		

Raw and processed RNA-seq data	This study	GEO: GSE89843
MISO reference files	(Katz et al., 2010)	https://miso.readthedocs.io/en/fastmiso/annotation.html
RBP reference table	(Ray et al., 2013)	Supplementary Data 2 in Ray et al. (2013)

**Software and Algorithms**		

SAS statistical software module (version 13.0.0)	JMP	https://www.jmp.com/en_us/home.html
Trimmomatic (version 0.22)	(Bolger et al., 2014)	http://www.usadellab.org/cms/?page=trimmomatic
STAR (version 2.3.0)	(Dobin et al., 2013)	https://github.com/alexdobin/STAR
HTSeq (version 0.6.1)	(Anders et al., 2014)	http://www-huber.embl.de/HTSeq/doc/overview.html
Picardtools (version 1.115)	Broad Institute, USA	https://broadinstitute.github.io/picard/
Samtools (version 1.115)	(Li et al., 2009)	http://samtools.sourceforge.net
Bedtools (version 2.17.0)	(Quinlan and Hall, 2010)	http://bedtools.readthedocs.io/en/latest/
MISO (version 0.5.3)	(Katz et al., 2010)	https://miso.readthedocs.io/en/fastmiso/
MATLAB (version R2015b)	The MathWorks Inc., USA	https://nl.mathworks.com/products/matlab.html
R (version 3.3.0)	(R Core Team, 2016)	https://www.r-project.org
R-studio (version 0.99.902)	(RStudio, 2016)	https://www.rstudio.com
Bioconductor package edgeR (version 3.12.1)	(Robinson and Oshlack, 2010)	https://bioconductor.org/packages/release/bioc/html/edgeR.html
Bioconductor package EDASeq (version 2.4.1)	(Risso et al., 2011)	http://bioconductor.org/packages/release/bioc/html/EDASeq.html
Bioconductor package PPSO (version 0.9-9991)	(Tolson and Shoemaker, 2007)	https://www.rforge.net/ppso/
Bioconductor package RUVSeq (version 1.4.0)	(Risso et al., 2014)	http://bioconductor.org/packages/release/bioc/html/RUVSeq.html
R-package e1071 (version 1.6-7)	CRAN	https://cran.r-project.org/web/packages/e1071/index.html
R-package Caret (version 6.0-71)	CRAN	https://cran.r-project.org/web/packages/caret/index.html
R-package Optunity (version 1.0)	STADIUS lab	http://optunity.readthedocs.io/en/latest/
R-package pROC (version 1.8)	CRAN	https://cran.r-project.org/web/packages/pROC/index.html
R-package ROCR (version 1.0-7)	CRAN	https://cran.r-project.org/web/packages/ROCR/index.html
R-package PAGODA (version 1.99.1)	(Fan et al., 2016)	http://hms-dbmi.github.io/scde/index.html
R-package Seqinr (version 3.3-3)	CRAN	https://cran.r-project.org/web/packages/seqinr/index.html
R-package VennDiagram (version 1.6.17)	CRAN	https://cran.r-project.org/web/packages/VennDiagram/index.html

### Contact for Reagent and Resource Sharing

Further information and requests for resources and reagents should be directed to and will be fulfilled by the Lead Contact Thomas Wurdinger (t.wurdinger@vumc.nl).

### Experimental Model and Subject Details

#### Clinical Sample Collection

Peripheral whole blood was drawn by venipuncture from cancer patients, patients with inflammatory and other non-cancerous conditions, and asymptomatic individuals at the VU University Medical Center, Amsterdam, The Netherlands, the Netherlands Cancer Institute (NKI/AvL), Amsterdam, The Netherlands, the Academic Medical Center, Amsterdam, The Netherlands, the Utrecht Medical Center, Utrecht, The Netherlands, the University Hospital of Umeå, Umeå, Sweden, the Hospital Germans Trias i Pujol, Barcelona, Spain, The University Hospital of Pisa, Pisa, Italy, and Massachusetts General Hospital, Boston, USA (see also Table S1). Whole blood was collected in 4-, 6-, or 10-mL EDTA-coated purple-capped BD Vacutainers containing the anti-coagulant EDTA. Samples for both training, evaluation, and independent validation cohorts were collected and processed similarly and simultaneously.

#### Clinical Data Annotation and Cohort Selection

Cancer patients were diagnosed by clinical, radiological and pathological examination, and were confirmed to have at moment of blood collection detectable tumor load. The NSCLC cohort includes 1 stage I, 2 stage II, 54 stage III, and 344 stage IV NSCLC samples (n=1 unknown stage, included in the metastasized late-stage cohort). For collection and annotation of clinical data, patient records were manually queried for demographic variables, i.e. patient age, gender, smoking, type of tumor, metastases, details of current and prior treatments, and co-morbidities. In case of transgender individuals, the new gender was stated (n=1). Platelet samples were collected before start of (a new) treatment or during treatment. A total of 106 NSCLC samples included were follow-up samples of the same patient (n=77 unique patients), of which 49 had two or more samples randomly assigned to the evaluation and/or validation cohort of the full cohort analysis, collected weeks to months after the first blood collection. Age-matching was performed retrospectively using a custom script in MATLAB, iteratively matching samples by excluding and including non-cancer and NSCLC samples aiming at a similar median age and age-range between both groups. Asymptomatic individuals were at the moment of blood collection, or previously, not diagnosed with cancer, but were not subjected to additional tests confirming the absence of cancer. The patients with inflammatory or other non-cancerous conditions did not have a diagnosed malignant tumor at the moment of blood collection. This study was conducted in accordance with the principles of the Declaration of Helsinki. Approval for this study was obtained from the institutional review board and the ethics committee at each participating hospital. Participants signed informed consent for blood collection and blood platelet analysis. Clinical follow-up of asymptomatic individuals is not available due to anonymization of these samples according to the ethical rules of the hospitals.

### Method Details

#### Blood Processing and Platelet Isolation

Whole blood samples in 4-, 6-, or 10-mL EDTA-coated Vacutainer tubes were processed using standardized protocols within 48 hours (Best et al., 2015, Nilsson et al., 2011). Whole blood collected in the VU University Medical Center, the Netherlands Cancer Institute, the Utrecht Medical Center, the University Hospital of Umeå, the Hospital Germans Trias i Pujol, and the University Hospital of Pisa was subjected to platelet isolation within 12 hours after blood collection. Whole blood samples collected at Massachusetts General Hospital Boston and the Academical Medical Center Amsterdam were stored overnight and processed after 24 hours. Platelet counts were obtained from the clinical records and quantified using the Sysmex XN9000 (Etten-Leur, NL) platelet quantification method. To isolate platelets, platelet rich plasma (PRP) was separated from nucleated blood cells by a 20-minute 120xg centrifugation step, after which the platelets were pelleted by a 20-minute 360xg centrifugation step. Removal of 9/10^th^ of the PRP has to be performed carefully to reduce the risk of contamination of the platelet preparation with nucleated cells, pelleted in the buffy coat. Centrifugations were performed at room temperature. Platelet pellets were carefully resuspended in RNAlater (Thermo Scientific) and after overnight incubation at 4°C frozen at -80°C.

#### Flow Cytometric Platelet Activation Analysis

To assess the relative platelet activation during our platelet isolations, we measured the surface protein expression levels of the constitutively expressed platelet marker CD41 (APC anti-human, clone: HIP8, Biolegend, cat nr. 303710) and platelet activation-dependent markers P-selectin (CD62P, PE anti-human, clone: AK4, Biolegend, cat nr. 304906) and CD63 (FITC anti-human, clone: H5C6, Biolegend, cat nr. 353006), using a BD FACSCalibur flow cytometer. We collected five 6-mL EDTA-coated Vacutainers tubes from each of six healthy donors, and determined the platelet activation state at baseline (0 hours), 8 hours, 24 hours, 48 hours, and 72 hours. As a negative control, we isolated at time point zero platelets from whole blood using a standardized platelet isolation protocol from citrate-anticoagulated whole blood that has been validated for inducing minimal platelet activation. This protocol consisted of a step of OptiPrep (Sigma-Aldrich, cat nr. D1556) density gradient centrifugation (350xg for 15 minutes) after collection of platelet rich plasma. This was followed by two washing steps first with Hepes, followed by a washing step in SSP+ buffer (Macopharma). We used 400 nM prostaglandin I2 (Sigma-Aldrich) before every centrifugation step to prevent platelet activation during the isolation procedure. As a positive control, we included platelets activated by 20 μM TRAP (TRAPtest, Roche, cat nr. 06675883190). Platelet pellets were after isolation prefixed in 0.5% formaldehyde (Roth), stained, and stored in 1% formaldehyde for flow cytometric analysis. Relative activation and mean fluorescent intensity (MFI) values were analyzed with FlowJo. Hence, absence of platelet activation during blood collection and storage was confirmed by stable levels of P-selectin and CD63 platelet surface markers.

#### RNA-seq Library Preparation

Preparation of samples for sequencing was performed in batches, and included per batch a mixture of clinical conditions. All samples have been subjected to the identical standardized thromboSeq protocol, including SMARTer cDNA amplification. For platelet RNA isolation, frozen platelets were thawed on ice and total RNA was isolated using the mirVana miRNA isolation kit (Ambion, Thermo Scientific, cat nr. AM1560). Platelet RNA was eluated in 30 μL elution buffer. We evaluated the platelet RNA quality using the RNA 6000 Picochip (Bioanalyzer 2100, Agilent), and included as a quality standard for subsequent experiments only platelet RNA samples with a RIN-value >7 and/or distinctive rRNA curves. All Bioanalyzer 2100 quality and quantity measures were collected from the automatically generated Bioanalyzer result reports using default settings, and after critical assessment of the reference ladder (quantity, appearance, and slope). The Truseq cDNA labeling protocol for Illumina sequencing (see below) requires ∼1 μg of input cDNA. Since a single mature platelet contains an estimated ∼2 femtogram of RNA (Teruel-Montoya et al., 2014), assuming an average platelet count of 300x10^6^ per mL of whole blood and highly efficient platelet isolation and RNA extraction, the estimated optimal yield of platelets from clinically relevant blood volumes (6 mL) is ∼3.6 μg. The average total RNA obtained from our blood samples is 146 ng (standard deviation: 130 ng, n=237 samples). To have sufficient platelet cDNA for robust RNA-seq library preparation, the samples were subjected to cDNA synthesis and amplification using the SMARTer Ultra Low RNA Kit for Illumina Sequencing v3 (Clontech, cat. nr. 634853). Prior to amplification, all samples were diluted to ∼500 pg/μL total RNA and again the quality was determined and quantified using the Bioanalyzer Picochip. For samples with a stock yield below 400 pg/μL, a volume of two or more microliters of total RNA (up to ∼500 pg total RNA) was used as input for the SMARTer amplification. Quality control of amplified cDNA was measured using the Bioanalyzer 2100 with DNA High Sensitivity chip (Agilent). All SMARTer cDNA synthesis and amplifications were performed together with a negative control, which was required to be negative by Bioanalyzer analysis. Samples with detectable fragments in the 300-7500 base pair (bp) region were selected for further processing. To measure the average cDNA length, we selected in the Bioanalyzer software the region from 200-9000 base pairs and recorded the average length. For labeling of platelet cDNA for sequencing, all amplified platelet cDNA was first subjected to nucleic acid shearing by sonication (Covaris Inc) and subsequently labeled with single index barcodes for Illumina sequencing using the Truseq Nano DNA Sample Prep Kit (Illumina, cat nr. FC-121-4001). To account for the low platelet cDNA input concentration, all bead clean-up steps were performed using a 15-minute bead-cDNA binding step and a 10-cycle enrichment PCR. All other steps were according to manufacturers protocol. Labeled platelet DNA library quality and quantity was measured using the DNA 7500 chip or DNA High Sensitivity chip (Agilent). To correlate total RNA input for SMARTer amplification, SMARTer cDNA yield, and Truseq cDNA yield, all samples with matched data available were subjected to a Pearson’s correlation test (cor.test-function in R). High-quality samples with product sizes between 300-500 bp were pooled (12-19 samples per pool) in equimolar concentrations for shallow thromboSeq and submitted for 100 bp Single-Read sequencing on the Illumina Hiseq 2500 platform using version 4 sequencing reagents. Precise and accurate quantification of the barcoded sample libraries and careful equimolar pooling is required to obtain equal total sequencing reads counts for all samples. For the deep thromboSeq experiment, we pooled 12 identically prepared platelet samples, and sequenced the same pool on four lanes of a Hiseq 2500 flowcell. Subsequently, four separate FASTQ-files per sample were merged in silico.

### Quantification and Statistical Analysis

#### Confounding Variable Analysis

To estimate the contribution of the variables 1) patient age (in years) at moment of blood collection, 2) whole blood storage time, 3) gender, and 4) smoking (current, former, never), we summarized the available data from Tables S1A–S1C and Figure S2C of our previous study (Best et al., 2015), and performed logistic regression analyses in the statistical software module SAS (v.13.0.0). Blood storage time was defined as the time between blood collection and the start of platelet isolation by differential centrifugation, stratified into a <12 hours group and a >12 hours group. For variables of samples of which data was missing, that particular value of the particular samples was excluded from the calculation. The joint predictive power of patient age, blood storage time, and the predictive strength of the diagnostics classifier for NSCLC, was assessed using a measure of logistic regression with nominal response, by selecting disease state as the Role Variable Y, and adding patient age, blood storage time, gender, smoking, and predictive strength for NSCLC as the model effects. All additional settings were set at default.

#### Processing of Raw RNA-Sequencing Data

Raw RNA-seq data of platelets encoded in FASTQ-files were subjected to a standardized RNA-seq alignment pipeline, as described previously (Best et al., 2015). Here, RNA-seq reads were subjected to 5’-end quality trimming and clipping of sequence adapters by Trimmomatic (version 0.22) (Bolger et al., 2014), mapped to the human reference genome (hg19) using STAR (version 2.3.0) (Dobin et al., 2013). Read summarization of only reads spanning introns (intron-spanning reads) was performed with HTSeq (version 0.6.1), using union intersection of uniquely aligned reads, which was guided by the Ensembl gene annotation version 75 (Anders et al., 2014). All subsequent statistical and analytical analyses were performed in R (version 3.3.0) and R-studio (version 0.99.902). Of samples that yielded less than 0.2x10^6^ intron-spanning reads in total after sequencing, we again sequenced the original Truseq preparation of the sample and merged the read counts generated from the two individual FASTQ-files after HTSeq count summarization (performed for n=52 samples). As expected, after sequencing of polyadenylated RNA we measured a significant enrichment of platelet sequence reads mapping to exonic regions. Sample filtering was performed by assessing the library complexity, which is partially associated with the intron-spanning reads library size. First, we excluded the genes that yielded <30 intron-spanning reads in >90% of the cohort for all platelet samples that were sequenced (n=784 Total, n=379 non-cancer and n=405 NSCLC, Figure S1G). This resulted in this platelet RNA-seq library in 4,722 different genes detected with sufficient coverage. For each sample, we quantified the number of genes for which at least one intron-spanning read was mapped, and excluded samples with <750 detected genes. Hereby we excluded two samples (n=0 (0% of total) non-cancer, n=2 (0.5% of total) NSCLC). Next, to exclude platelet samples that show low intersample correlation, we performed a leave-one-sample-out cross-correlation analysis. Following data normalization (see section ‘Data Normalization and RUV Factor Correction’), for each sample in the cohort, all samples except the ‘test sample’ were used to calculate the median counts-per-million expression for each gene (reference profile). Following, the comparability of the test sample to the reference set was determined by Pearson’s correlation. Samples with a correlation <0.5 were excluded (n=3), and the remaining 779 samples were included in this study. Of note, we observed delicate differences in the Bioanalyzer cDNA profiles (spiked/smooth patterns), irrespective of patient group, but with a significant correlation to average cDNA length. We measured the average length of concatenated reads mapped to intergenic regions for spiked and smooth samples separately using Bedtools (version 2.17.0, Bedtools merge following Bedtools intersection), and observed that the majority of reads (>10.9% for spiked samples and >13.5% for smooth samples, n=50 samples each) had an average fragment length (concatenated reads) of <250 nt, with a peak at 100-200 nt. We attribute the differences in cDNA profiles at least partly to ‘contaminating’ plasma DNA retained during the platelet isolation procedure. To prevent potential plasma DNA from contributing to our computational platelet RNA analyses, we only selected spliced intron-spanning RNA reads. Practically, sequestration of sequencing reads by ‘contaminating’ plasma DNA reduces the number of reads available for RNA-sequencing analysis. A detailed overview of algorithm settings is provided in Table S3.

#### Assessment Technical Performance thromboSeq

We observed in the platelet RNA a rich repertoire of spliced RNAs, including 4,000-5,000 different messenger and non-coding RNAs. To estimate the efficiency of detecting the repertoire of 4,000-5,000 platelet RNAs from ∼500 pg of total platelet RNA input, we summarized all gene tags with at least 30 non-normalized intron-spanning read counts. We investigated whether collection of more Single-Read 100 bp RNA-seq reads (∼5x deeper: deep thromboSeq) of the platelet cDNA libraries (n=12 healthy donors) yielded in detection of more low-abundant RNAs. For this, we selected the gene tags that had more than 10 raw intron-spanning reads in at least one sample. This was performed separately for shallow and deep thromboSeq. For visualization purposes, we calculated the median raw intron-spanning read counts, log_2_-transformed the counts (after adding one count to all tags), and plotted the 20,000 gene tags with highest count numbers. Again, this was performed separately for shallow and deep thromboSeq data. Increasing the average coverage of shallow thromboSeq ∼5x does not yield in significantly enriched detection of low-abundant platelet genes. For all boxplots presented in the manuscript, the box indicates the interquartile range (IQR), the horizontal black line indicates the median values, and the whiskers range 1.5 x the IQR.

#### Differential Splicing Analysis

Prior to differential splicing analyses the data was subjected to the iterative correction-module as described in the section ‘Data Normalization and RUV Factor Correction’ (age correlation threshold 0.2, library size correlation threshold 0.8). Corrected read counts were converted to counts-per-million, log_2_-transformed, and multiplied by the TMM-normalization factor calculated by the calcNormFactors-function of the R-package edgeR (Robinson et al., 2010). For generation of differential spliced gene sets, the after fitting of negative binominal models and both common, tag-wise and trended dispersion estimates were obtained, differentially spliced transcripts were determined using a generalized linear model (GLM) likelihood ratio test, as implemented in the edgeR-package. For data signal purposes, we performed differential splicing analyses with post-hoc gene ontology interpretation using the corrected read counts as input for differential splicing analyses, whereas for reproducibility of the data during classification tasks we used the non-corrected raw read counts as input. Genes with less than three logarithmic counts per million (logCPM) were removed from the spliced RNA gene lists. RNAs with a p value corrected for multiple hypothesis testing (FDR) below 0.01 were considered as statistically significant. Unsupervised hierarchical clustering of heatmap row and column dendrograms was performed by Ward clustering and Pearson distances. Non-random partitioning and the corresponding p value of unsupervised hierarchical clustering was determined using a Fisher’s exact test (fisher.test-function in R). To determine differentially splicing levels between platelets of non-cancer individuals and NSCLC patients, we included only samples assigned to the patient age, smoking status and blood storage time-matched cohort (training, evaluation and independent validation, n=263 in total).

#### Analysis of RNA-seq Read Distribution

Distribution of mapped RNA-seq reads of platelet cDNA, and thus the origin of the RNA fragments, was investigated in samples assigned to the patient age, smoking status and blood storage time-matched NSCLC/non-cancer cohort (training, evaluation, and independent validation, totaling 263 samples). The mitochondrial genome and human genome, of which the latter includes exonic, intronic, and intergenic regions were quantified separately. Read quantification was performed using the Samtools View algorithm (version 1.2, options -q 30, -c enabled). For quantification of exonic reads, we only selected reads that mapped fully to an exon by performing a Bedtools Intersect filter step (-abam, -wa, -f 1, version 2.17.0) prior to Samtools View quantification. We used bed-files of exonic, intronic, and intergenic regions annotated in Ensembl gene annotation version 37 and hg19 as a reference. Spliced RNAs were filtered from the aligned reads by selection of a cigar-tag in the bam-file, and reads mapping to the mitochondrial genome were selected by only quantifying reads mapping to ‘chrM’. We determined the ratios of reads mapping to the specific genomic regions by calculating the proportion of reads as compared to the total number of quantified reads per sample. Independent two-sided Student’s t-test was performed using the t.test-function in R.

#### Alternative Isoforms and Exon Skipping

We employed the MISO algorithm (Katz et al., 2010) for alternative splicing analysis in our 100 bp Single-Read RNA-seq data. Briefly, the MISO algorithm quantifies the number of reads favouring inclusion or exclusion of a particular annotated event, such as exon skipping, or RNA isoforms. By scoring reads supporting either one variant or the other (on/off) and scoring reads supporting both isoforms, the algorithm infers the ratio of inclusion, and thereby the percent spliced in (PSI).

##### Processing of RNA-seq Data for MISO Splicing Analysis

For the MISO RNA splicing analyses, FASTQ-files of the patient age, smoking status and blood storage time-matched NSCLC/non-cancer cohort were again subjected to Trimmomatic trimming and clipping, and STAR read mapping (see also section ‘Processing of Raw RNA-Sequencing Data’). To create an uniform read length of all inputted reads, as required by the MISO algorithm, trimmed reads were cropped to 92 nt and reads below a read length of 92 nt were excluded from analysis. After addition of read groups using Picard tools (AddOrReplaceReadGroups-function, version 1.115), MISO sam-to-bam conversion was performed, and the indexed bam files were subjected to the MISO algorithm (version 0.5.3) using hg19 and the indexed Ensembl gene annotation version 65 as reference. MISO output files were summarized using the summarize_miso-function. Summarized MISO files of isoforms and skipped exons were subsequently converted into ‘psi’ count matrices and ‘assigned counts’ count matrices using a custom script in MATLAB. A detailed overview of algorithm settings is provided in Table S3.

##### Identification of Alternatively Spliced Isoforms

For alternative isoform analysis, we narrowed the analysis to the 4,722 genes identified with confident intron-spanning expression levels in platelets (see also section ‘Processing of Raw RNA-Sequencing Data’). For each annotated Ensemble transcript ID, available in the MISO summary output files, the assigned read counts (reads assigned to the particular RNA isoform) were summarized in a count matrix. To ensure proper detection of the isoform, we excluded RNA isoforms with <10 reads in >90% of the sample cohort, and applied TMM- and counts-per-million normalization. Next, differential expression analysis among annotated Ensembl transcripts was performed, and the most significant hits (FDR<0.01, logCPM>1) were selected. For details regarding the differential expression analysis, see section ‘Differential Splicing Analysis‘. For identification of multiple RNA isoforms per parent gene locus, we matched the Ensembl transcript IDs (enst) with Ensembl gene IDs (ensg) and calculated the frequency metrics of the ensg-tags for the significant enst-tags (Figure 3A left pie chart). Distribution of alternatively spliced isoforms was assessed by including all enst-tags per parent gene locus, and comparing the median expression values for both non-cancer and NSCLC samples. Isoforms that showed in all cases increased or decreased levels were scored as non-alternatively spliced. Isoforms that exhibited enrichment in either group but a decrease in the other, and the opposite for at least one other isoform were scored as alternatively spliced RNAs (Figure 3A right pie chart).

##### Identification of Exon Skipping Events

For analysis of exon skipping events, we developed a custom analysis pipeline summarizing reads supporting inclusion or exclusion of annotated exons and scoring the relative contribution in groups of interest, i.e. non-cancer versus NSCLC. The input for the algorithm is a PSI-values count matrix and an ‘assigned counts’ count matrix, as generated from summary output files generated by MISO. The former count matrix is required to calculate the relative PSI-values and distribution per group, the latter count matrix is required to only include exons with sufficient coverage in the RNA-seq data (i.e. >10 reads in >60% of the samples, which support both inclusion (1,0) and exclusion (0,1) of the variant, see also Katz et al.). The coverage selector downscaled the available exons for analysis to 230 exons. To select differential levels of exon skipping events, *PSI*-values were compared among non-cancer and NSCLC using an independent two-sided Student’s t-test including post-hoc false discovery rate (FDR) correction (t.test- and p.adjust-function in R). Events with an FDR<0.01 were considered as potential skipped exon events. The ΔPSI-value was calculated by subtracting per skipping event the median PSI-value of non-cancer from the median PSI-value NSCLC.

#### P-selectin Signature

To determine the correlation between P-selectin levels and exonic read counts, we compared the P-selectin (SELP, ENSG00000174175) counts-per-million values of 263 patient age, smoking status and blood storage time-matched individuals to the number of exon-mapped reads. P-selectin expression levels were collected from log_2_-transformed, TMM-normalized, and counts-per-million transformed read counts, subjected to RUV-mediated correction (see section ‘Data Normalization and RUV Factor Correction’, age correlation threshold 0.2, library size correlation threshold 0.9). Exonic read counts to P-selectin expression levels correlation analysis was performed using Pearson’s correlation. To identify gene expression correlated to P-selectin enrichment, we calculated Pearson’s correlations of all individual genes (n=4,722 in total) to the P-selectin expression levels. Data was summarized in a histogram, and we compiled a P-selectin signature by selecting positively (r>0) and most significantly (FDR<0.01, adjusted for multiple hypothesis testing) correlated genes. The P-selectin signature was compared with all differentially and increasingly spliced genes between non-cancer and NSCLC, and summarized in a Venn diagram (VennDiagram-package in R).

#### RBP-thromboSearch Engine

To identify RNA-binding protein (RBP) profiles associated with the TEP signatures in NSCLC patients, we designed and developed the RBP-thromboSearch engine. The rationale of this algorithm is that enriched binding sites for particular RBPs in the untranslated regions (UTRs) of genes is correlated to stabilization or regulation of splicing of that specific RNA. The algorithm identifies the number of matching RBP binding motifs in the genomic UTR sequences of genes confidently identified in platelets. Subsequently, it correlates for each included RBP the n binding sites to the logarithmic fold-change (logFC) of each individual gene, and significant correlations are ranked as potentially involved RBPs. For this analysis, we collected previously well-characterized RBP binding motifs from literature (Ray et al., 2013). The algorithm exploits the following assumptions: 1) more binding sites for a particular RBP in a UTR region predicts increased regulation of the gene either by stabilization or destabilization of the pre-mRNA molecule (Oikonomou et al., 2014), 2) the functions in 1) are primarily driven by a single RBP and not in combinations or synergy with multiple RBPs or miRNAs, or other cis or trans regulatory elements, and 3) the included RBPs are present on protein level in platelets of non-cancer individuals and/or NSCLC patients. In order to determine the n RBP binding sites-logFC correlations, the algorithm performs the following calculations and quality measure steps:

(i) The algorithm selects from all inputted genes the annotated RNA isoforms and identifies genomic regions of the annotated RNA isoforms that are associated with either the 5’-UTR or 3’-UTR. The genomic coding sequence is extracted from the human hg19 reference genome using the getfasta-function in Bedtools (version 2.17.0). For this study, we used the Ensembl annotation version 75.
(ii) All characterized motif sequences extracted from literature (102 in total, Table S3 of Ray et al. (Ray et al., 2013), filtered for Homo Sapiens) are reduced to 547 non-redunant (‘A’, ‘G’, ‘C’, and ‘T’-sequence) annotations according to the IUPAC motif annotation. These non-redundant motif sequences serve as the representative motif sequences for the initial search.
(iii) In an iterative manner, per RBP the associated non-redundant RBP motif sequences are matched with all identified and included UTR sequences (using the str_count-function of the seqinr package in R).
(iv) The algorithm identifies the number of reads mapping to each UTR region per sample using Samtools View (q 30, -c enabled). UTR sequences with no or minimal coverage were considered to be non-confident for presence in platelets. To account for the minimal bias introduced by oligo-dT-primed mRNA amplification (Ramsköld et al., 2012), we set the threshold of number of reads for the 3’-UTR at five reads, and for the 5’-UTR at three reads.
(v) For all 5’- and 3’-UTRs with sufficient coverage associated with the same parent gene (ensg), all matched UTR-non-redundant motif hits were summed, and summarized in a gene-motif matrix. Non-redundant motifs were converted to RBP-ids by overlaying all possible RBP-motif matches. This matrix is used for downstream analyses, data interpretation, and visualization.

Correlations between logFC and n RBP binding sites were determined for all RBPs using Pearson’s correlation, and summarized in a volcano plot.

#### Data Normalization and RUV Factor Correction

To reduce the influence of confounding factors participating in the classification model, we applied the following approach for iterative RNA-sequencing data correction. The correction module is based on the remove unwanted variation (RUV) method, proposed by Risso et al. (Peixoto et al., 2015, Risso et al., 2014), supplemented by selection of ‘stable genes’ (independent of the confounding variables), and an iterative and automated approach for removal and inclusion of respectively unwanted and wanted variation. The RUV correction approach exploits a generalized linear model, and estimates the contribution of covariates of interest and unwanted variation using singular value decomposition (Risso et al., 2014). In principle, this approach is applicable to any RNA-seq dataset and allows for investigation of many potentially confounding variables in parallel. Of note, the iterative correction algorithm is agnostic for the group to which a particular sample belongs, in this case NSCLC or non-cancer, and the necessary stable gene panels are only calculated by samples included in the training cohort. The algorithm performs the following multiple filtering, selection, and normalization steps, i.e.:

(i) Filtering of genes with low abundance, i.e. less than 30 intron-spanning spliced RNA reads in more than 90% of the sample cohort (included in the main data processing pipeline, see section ‘Processing of Raw RNA-Sequencing Data’).
(ii) Determination of genes showing least variability among confounding variables (‘stable genes’). For this, the non-normalized raw reads counts of each gene that passed the initial filter in (i) were correlated using Pearson’s correlation to either the total intron-spanning library size (as calculated by the DGEList-function of the edgeR package in R) or the age of the individuals. In case of correlations towards the intron-spanning library size, genes with a high Pearson correlation (towards 1) show the least variability after counts-per-million normalization, and were thus designated as stable genes.
(iii) Raw read counts of the training cohort were subjected to the RUVg-function from the RUVSeq-package in R. The stable genes identified among the confounding variables were used as ‘negative control genes’. Following, the individual estimated factors for each sample identified by RUVg are correlated to potential confounding factors (in the current study: library size, age of the individual) or the group of interest (for example non-cancer versus NSCLC). The continuous (confounding) variables are correlated to the estimated variance of the samples. Dichotomous variables (e.g. group) are compared using a two-sided, independent Student’s t-test. In both instances, the p value was used as a significance surrogate between the RUVg variable and the (confounding) variable. Of note, to prevent removal of a variable likely correlated to group, we applied two rules prior to matching a variable to a (confounding) factor, i.e. a) the p value between RUVg variable and group should be at least >1e-5, and b) the p value between RUVg variable and the other variable should be at least <0.01. Raw non-normalized reads were corrected for RUVg variable x in case this variable was correlated to a confounding factor. Finally, the total intron-spanning library size per sample was adjusted by calculating the sum of the RUVg-corrected read counts per sample.
(iv) RUVg-normalized read counts are subjected to counts-per-million normalization, log_2_-transformation, and multiplication using a TMM-normalization factor. The latter normalization factor was calculated using a custom function, implemented from the calcNormFactors-function in the edgeR package in R. Here, the eligible samples for TMM-reference sample selection can be narrowed to a subset of the cohort, i.e. for this study the samples assigned to the training cohort, and the selected reference sample was locked.

We applied this iterative correction module to all analyses in this work. The estimated RUVg number of factors of unwanted variation (k) was 3. We directly compared the performance of our previous normalization module and the iterative correction module presented in this study using relative log intensity (RLE) plots, and observed superior removal of variation within the expression data. RLE-plots were generated using the plotRLE-function of the EDASeq package. Significance of the reduction of inter-sample variability was determined by calculating the absolute difference of the samples’ median RLE counts to the overall median RLE counts for all samples for each sample with and without RUV-mediated factor correction.

#### PSO-Enhanced SVM Algorithm Development

The PSO-enhanced thromboSeq algorithm implements multiple improvements over the previously published thromboSeq algorithm (Best et al., 2015). First, we improved algorithm optimization and training evaluation by implementing a training-evaluation approach. A total of 93 samples for the matched cohort and 120 samples for the full cohort assigned for training-evaluation were used as an internal training cohort. These samples served as reference samples for the iterative correction module (see ‘Data Normalization and RUV Factor Correction’-section), initial gene panel selection by a likelihood ratio ANOVA test (see ‘Differential Splicing Analysis’-section), SVM-parameter optimization, and final algorithm training and locking (selection of support vectors). Second, after the likelihood ratio ANOVA analysis we removed genes with high internal correlation (findCorrelations-function in the R-package caret), as these were previously suggested to contribute to unwanted noise in SVM-models. Third, we implemented a recursive feature elimination (RFE) algorithm, previously proposed by Guyon et al. (Guyon et al., 2002), to enrich the gene panels for genes most relevant and contributing to the SVM classifiers. Fourth, following the final SVM cost and gamma parameter grid search, we performed additional refinement of the cost and gamma parameters, by enabling an internal, second particle swarm optimization algorithm (cv.particle_swarm-function in the R-package Optunity). This internal particle swarm algorithm was employed to investigate and pinpoint neighboring values of the optimal gamma and cost parameters determined by the SVM grid search for more optimal internal SVM performance. Fifth, the entire SVM classification algorithm was subjected to a particle swarm optimization algorithm (PSO), implemented by the ppso-package in R (optim_ppso_robust-function) (Tolson and Shoemaker, 2007). Particle swarm intelligence is based on the position and velocity of particles in a search-space that are seeking for the best solution to a problem. Upon iterative recalibration of the particles based on its local best solution and overall best solution, a more refined estimate of the input parameters and algorithm settings can be achieved. The implemented algorithm allows for real-time visualization of the particle swarms, optimization of multiple parameters in parallel, and deployment of the iterative ‘function-calls’ using multiple computational cores, thereby advancing implementation of large classifiers on large-sized computer clusters. The PSO-algorithm aims to minimize the ‘1-AUC’-score. We employed for our matched NSCLC/non-cancer cohort classifier 100 particles with 10 iterations and for the full NSCLC/non-cancer cohort classifier 200 particles with 7 iterations. We optimized four steps of the generic classification algorithms, i.e. (i) the iterative correction module threshold used for selection of genes identified as stable genes among the library size, (ii) the FDR-threshold included in the differential splicing filter applied to the results of the likelihood-ratio ANOVA test, (iii) the exclusion of highly correlated genes selected after the likelihood ANOVA test, and (iv) number of genes passing the RFE-algorithm (see also Figure S4A). Predefined ranges were submitted to the PSO-algorithm for every classification task presented in the this study. Training of SVM algorithms was performed using a two-times internal cross validation, and an initial gamma and cost parameter range for the grid search of 2ˆ(-20:0) and 2ˆ(0:20) respectively. To account for undetected genes in the validation cohort, potentially hampering normalization of the data and reducing algorithm performance, genes with counts between zero and 12 (matched cohort) and 2 (full cohort) were replaced by the median counts of the training cohort for that particular gene.

#### Performance Measure PSO-Enhanced thromboSeq

We assessed the performance, stability, and reproducibility of the PSO-enhanced thromboSeq platform using multiple training, evaluation, and independent validation cohorts. All classification experiments were performed with the PSO-enhanced thromboSeq algorithm, using parameters optimized by particle swarm intelligence. We assigned for the matched cohort 133 samples for training-evaluation, of which 93 were used for RUV-correction, gene panel selection, and SVM training (training cohort), and 40 were used for gene panel optimization (evaluation cohort). The full cohort contained 208 samples for training-evaluation, of which 120 were used for RUV-correction, gene panel selection, and SMV training (training cohort), and 88 were used for gene panel optimization (evaluation cohort). All random selection procedures were performed using the sample-function as implemented in R. For assignment of samples per cohort to the training and evaluation cohorts, only the number of samples per clinical group was balanced, whereas other potentially contributing variables were not stratified at this stage (assuming random distribution among the groups). Following, an SVM model was trained using the training samples, and the samples assigned to the independent validation cohort were predicted. The late-stage NSCLC samples and early-stage locally advanced NSCLC samples were validated separately resulting in two ROC curves. The 53 locally advanced NSCLC samples were age-matched with 53 non-cancer individuals selected from the non-cancer samples of the independent validation cohort. Performance of the training cohort was assessed by a leave-one-out cross validation approach (LOOCV, see also (Best et al., 2015)). During a LOOCV procedure, all samples minus one (‘left-out sample’) are used for training of the algorithm. Each sample is predicted once, resulting in the same number of predictions as samples in the training cohort. The list of stable genes among the initial training cohort, determined RUV-factors for removal, and final gene panel determined by swarm-optimization of the training-evaluation cohort were used as input for the LOOCV procedure. As a control for internal reproducibility, we randomly sampled training and evaluation cohorts, while maintaining the validation cohorts and the swarm-guided gene panel of the original classifier, and perform 1000 (matched and full cohort NSCLC/non-cancer) training and classification procedures. As a control for random classification, class labels of the samples used by the SVM-algorithm for training of the support vectors were randomly permutated, while maintaining the swarm-guided gene list of the original classifier. This process was performed 1000 times for the matched and full NSCLC/non-cancer cohort classifiers. P values were calculated accordingly, as described previously (Best et al., 2015). Results were presented in receiver operating characteristics (ROC) curves, and summarized using area under the curve (AUC)-values, as determined by the ROCR-package in R. AUC 95% confidence intervals were calculated according to the method of Delonge using the ci.auc-function of the pROC-package in R.

#### Gene Ontology Analysis

For the gene ontology analysis, we investigated co-associated gene clusters using the PAGODA functions implemented in version 1.99 of the scde R-package (http://pklab.med.harvard.edu/scde/). PAGODA allows for clustering of redundant heterogeneity patterns and the identification of de novo gene clusters through pathway and gene set over-dispersion analysis (Fan et al., 2016). In particular, the ability to identify de novo gene clusters is of interest for the analysis of platelet RNA-seq data, as platelet biological functions are potentially unannotated and can only be inferred by unbiased cluster analysis. Gene IDs as selected by differential splicing analysis (n=1,622) were used as input to generate gene ontology library files. We used a distance threshold of 0.9 for the PAGODA redundancy reduction, and identification of de novo gene options was enabled. Remaining steps in the analysis were according to instructions from the PAGODA authors. PAGODA analysis revealed four major clusters (one existing and three de novo gene clusters) of co-regulated genes that were correlated to disease state. We selected clusters with a significantly enriched multiple hypothesis testing corrected z-score (adjusted z-score). The de novo clusters were further curated manually using the PANTHER Classification System (http://pantherdb.org/) on the 26^th^ of September 2016.

### Data and Software Availability

#### Data Resources

The raw sequencing data reported in this paper has been deposited into the NCBI GEO database under accession number GSE89843.

## Author Contributions

M.G.B. and T.W. designed the study and wrote the manuscript. M.M., A.-L.N.N., A.E.H.i.t.V., C.L., T.Y.L., L.L.M., G.K., E.G., J.C.R., S.I., J.K., M.H., S.C.d.J., R.T.U., I.E.H., G.P., J.G.-A., H.-J.B., D.P.N., W.P.V., D.v.d.B., B.Y., R.J.A.N., N.K., R.R., E.L.-L., K.B.L., B.A.T., A.J.d.L., E.F.S., and M.M.v.d.H. provided clinical samples and data. M.G.B., N.S., A.V., A.V.F., L.-A.T.K.F., F.R., P.S., H.V., E.P., L.E.W., J.B., M.E., L.W., F.F., J.D.S., J.T., and T.W. performed sample processing for RNA-seq. A.V. performed GO analyses. A.V.F. performed flow cytometry experiments. M.G.B., N.S., S.G.J.G.I.t.V., A.V., M.M., A.N.N., A.V.F., I.E.K., H.M.H., C.M., P.W., R.R., B.A.T., A.J.d.L., E.F.S., M.M.v.d.H., and T.W. performed data analyses and interpretation. All authors provided critical comments on the manuscript.

## Supplemental Information

Document S1. Figures S1–S4

Table S1. Patient Characteristics, Related to Figures 1 and S1

Table S2. Differentially Spliced Genes in Matched NSCLC/non-cancer Cohort, Related to Figure 2

Table S3. Settings of thromboSeq Data Processing Software, Related to Figure 6 and STAR Methods

Document S2. Article plus Supplemental Information
