# Supplementary material for: Swarm Intelligence-Enhanced Detection of Non-Small-Cell Lung Cancer Using Tumor-Educated Platelets
Source: Cancer Cell. 2017 Aug 14;32(2):238–252.e9. doi: 10.1016/j.ccell.2017.07.004 (PMC6381325; doi:10.1016/j.ccell.2017.07.004)
Supplement: Document S1. Figures S1–S4 [file mmc1.pdf]

Cancer Cell, Volume *32*

Supplemental Information

Swarm Intelligence-Enhanced Detection of Non-Small-Cell Lung Cancer Using Tumor-Educated Platelets

Myron G. Best, Nik Sol, Sjors G.J.G. In `t Veld, Adrienne Vancura, Mirte Muller, Anna-Larissa N. Niemeijer, Aniko V. Fejes, Lee-Ann Tjon Kon Fat, Anna E. Huis In `t Veld, Cyra Leurs, Tessa Y. Le Large, Laura L. Meijer, Irsan E. Kooi, François Rustenburg, Pepijn Schellen, Heleen Verschueren, Edward Post, Laurine E. Wedekind, Jillian Bracht, Michelle Esenkbrink, Leon Wils, Francesca Favaro, Jilian D. Schoonhoven, Jihane Tannous, Hanne Meijers-Heijboer, Geert Kazemier, Elisa Giovannetti, Jaap C. Reijneveld, Sander Idema, Joep Killestein, Michal Heger, Saskia C. de Jager, Rolf T. Urbanus, Imo E. Hoefer, Gerard Pasterkamp, Christine Mannhalter, Jose Gomez-Arroyo, Harm-Jan Bogaard, David P. Noske, W. Peter Vandertop, Daan van den Broek, Bauke Ylstra, R. Jonas A. Nilsson, Pieter Wesseling, Niki Karachaliou, Rafael Rosell, Elizabeth Lee-Lewandrowski, Kent B. Lewandrowski, Bakhos A. Tannous, Adrianus J. de Langen, Egbert F. Smit, Michel M. van den Heuvel, and Thomas Wurdinger

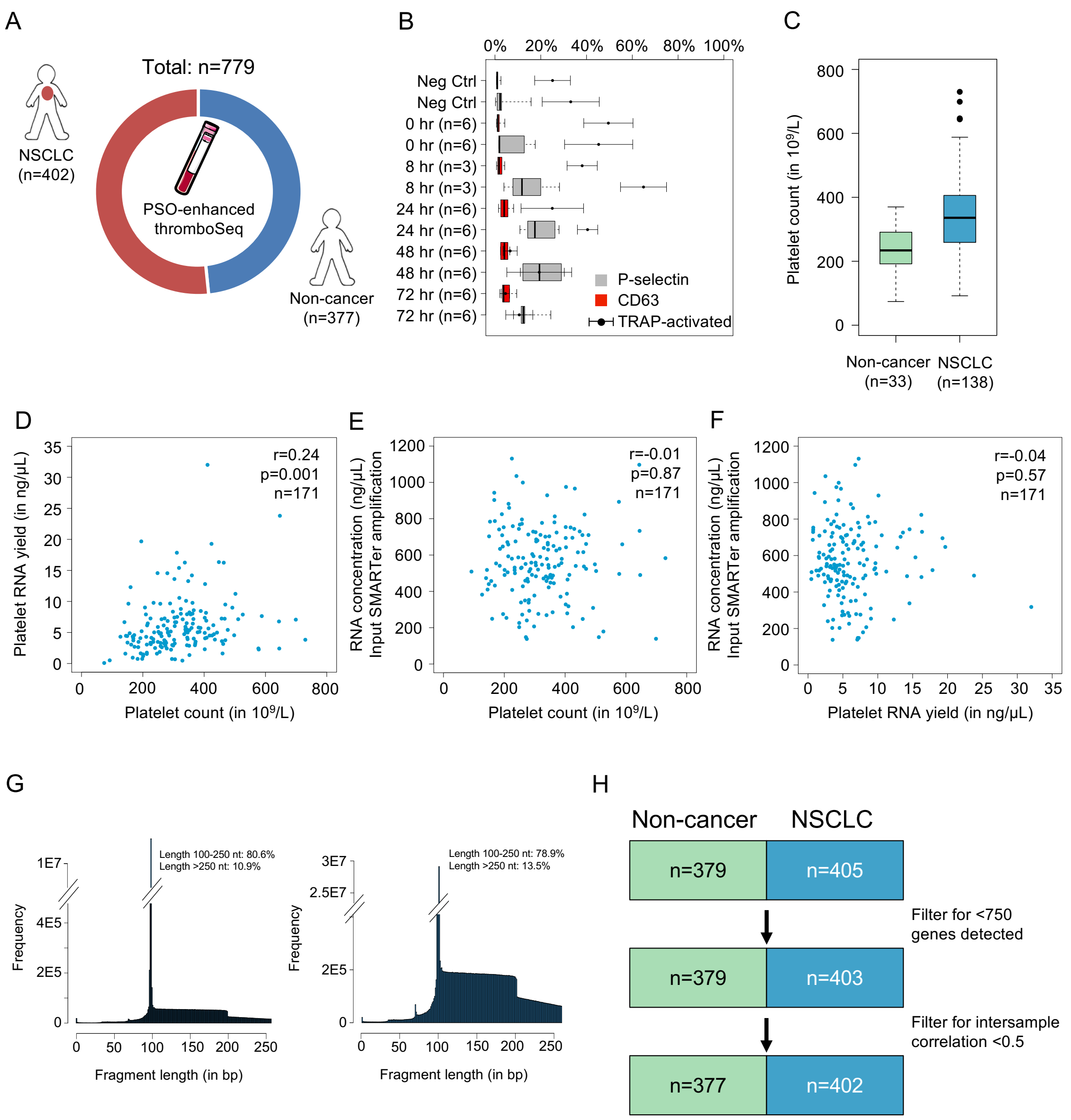

Figure S1, related to Figure 1. ThromboSeq pre-analytical evaluation.

(**A**) Overview of Non-cancer and NSCLC platelet samples (total of 779) included in this study for thromboSeq. (**B**) Overview of platelet activation markers as measured by flow cytometric analysis of n=3 (8 hour time point) or n=6 (other time points) platelet samples collected from healthy donors and isolated using the thromboSeq platelet isolation protocol. Gray and red boxes represent average percentage of platelets expressing respectively P-selectin or CD63 on the surface. The box indicates the interquartile range (IQR), black line represents the median, and the whiskers indicate 1.5 x IQR. Dots represent expression of these surface markers after platelet activation with TRAP. Platelet samples are only minimally activated using the thromboSeq platelet isolation protocol. Neg Ctrl = negative control; platelet samples isolated

according to an isolation protocol validated for minimal platelet activation. (**C**) Boxplot indicating the platelet counts of Non-cancer (n=33) and NSCLC (n=138) individuals of whom data was available. Platelet counting was performed on the day of blood collection or up to three days before blood collection. The box indicates the interquartile range (IQR), black line represents the median, and the whiskers indicate 1.5 x IQR. (**D**) Correlation plot of platelet count (x-axis) and the matching platelet RNA yield (y-axis). A moderate correlation was observed (r=0.24, p=0.001, n=171, Pearson's correlation). (**E**) Correlation plot of platelet count (x-axis) and the estimated RNA input for thromboSeq (y-axis). No significant correlation was observed (r=-0.01, p=0.87, n=171, Pearson's correlation). (**F**) Correlation plot of platelet RNA yield (x-axis) and the estimated RNA input for thromboSeq (y-axis). No significant correlation was observed (r=-0.04, p=0.57, n=171, Pearson's correlation). (**G**) Histogram of the average fragment length of reads mapped to intergenic regions for both spiked (left) and smooth (right) samples (n=50 samples each, randomly sampled from age, smoking, and blood storage time-matched cohort). The percentage of reads with specific concatenated fragment size are indicated in the individual plots. (**H**) Flowchart demonstrating sample filtering steps during pre-analytical bioinformatic quality control steps. Values in the boxes indicate sample numbers per group (green = 'Non-cancer', blue = 'NSCLC').

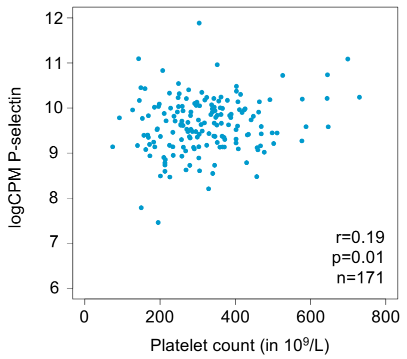

Figure S2, related to Figure 4. Correlation plot platelet count to P-selectin.

Correlation plot of platelet count (x-axis) to log_2-transformed counts-per-million (logCPM) of P-selectin in the patient age, smoking and blood storage time-matched cohort. A moderate correlation was observed (r=0.19, p=0.01, n=171, Pearson's correlation).

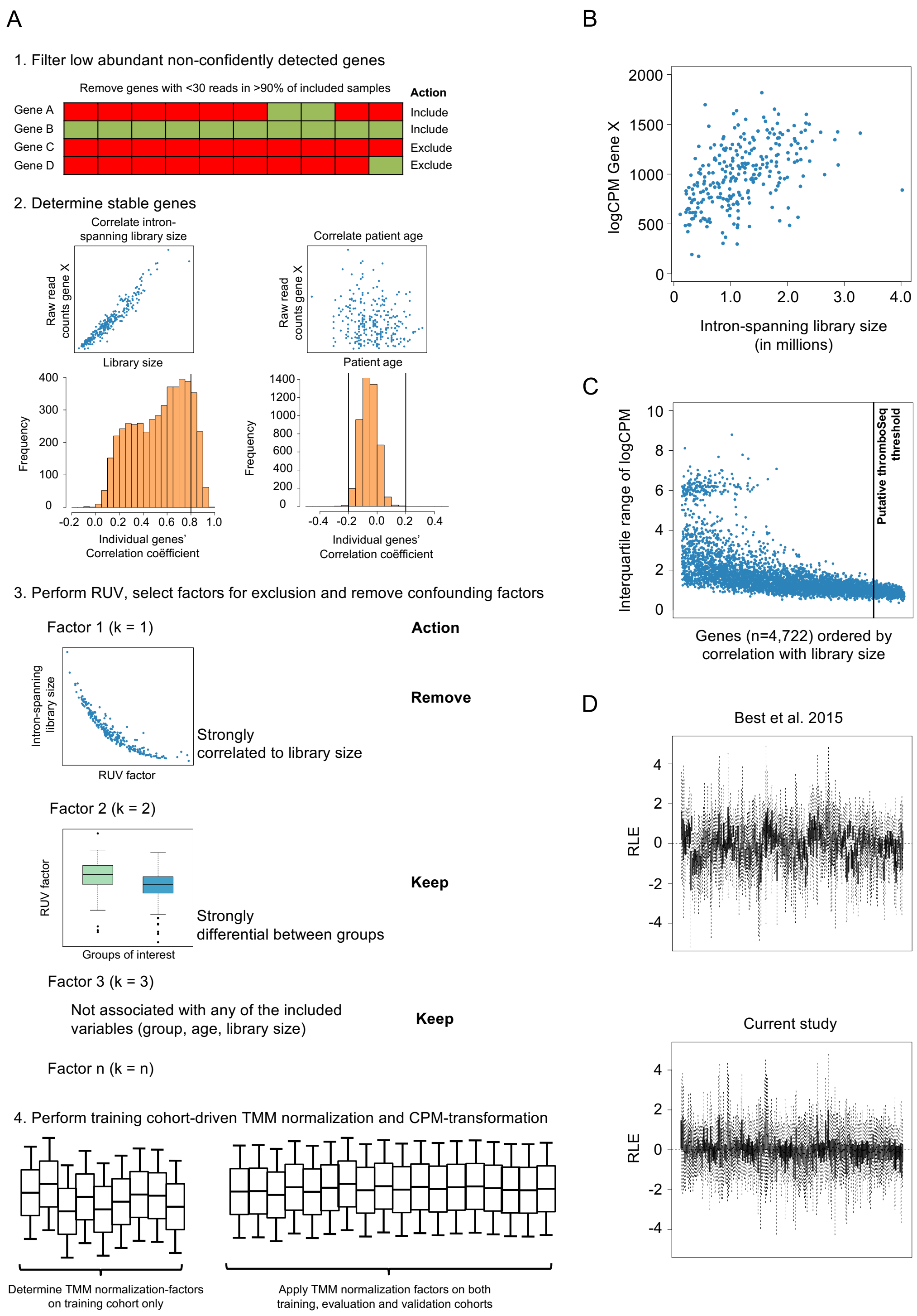

Figure S3, related to Figure 6. Schematic overview of RUV factor correction module for the PSO-enhanced thromboSeq classification algorithm.

(**A**) Schematic overview of the iterative correction module as implemented in thromboSeq. The RNA-seq data correction procedure includes multiple steps, i.e. 1) filtering of low abundant genes, 2) determination of stable genes among confounding variables, 3) raw-read counts Remove Unwanted Variation (RUV)-based factor

analysis and correction, and 4) reference group-mediated counts-per-million (CPM) and TMM-normalization**.** In detail, in step 1 genes with low confidence of detection, i.e. less than 30 intron-spanning spliced RNA reads in more than 90% of the sample cohort, are excluded. In the schematic example, the two upper genes (rows) contain in >90% of the samples (in this schematic example n=10 in total) sufficient numbers of reads, as indicated by the green boxes. Thus, these genes will be included for analysis. The lower two boxes indicate insufficient numbers of samples with sufficient numbers of genes, thus prompting the algorithm to remove these particular genes from the downstream analyses. Secondly, the algorithm searches for genes that show a stable expression pattern among all other samples. For this, the algorithm performs multiple Pearson's correlation analyses among a (potential confounding) variable and raw read counts, resulting in a distribution of the correlation coefficients. In the schematic figure, this is shown for intron-spanning reads library size (left) and patient age (right). The correlation distribution is shown below, and the putative thresholds (also subjected to PSO selection) are indicated by black lines. Of note, as the raw intron-spanning read counts are normalized by CPM normalization afterwards, stable genes have to approximate a correlation coefficient of one. During the third step, the algorithm first identifies factors contributing to the data in an unbiased way, using the RUVSeq-correction module (RUVg-function). The RUVSeq correction approach estimates and corrects based on a generalized linear model of a subset of genes and by singular value decomposition the contribution of covariates of interest and unwanted variation. Secondly, the algorithm iteratively correlates the variable of interest (group) and potentially confounding variables (patient age and blood storage time) to the factors identified by RUVSeq. If a factor is determined to be correlated to a confounding factor (e.g. intron-spanning reads library size in 'Factor 1'), the factor will be marked for removal ('Remove'). Alternatively, if a factor is determined to be correlated to the factor of interest (e.g. group in 'Factor 2') or to none of the factors identified as involved factors (e.g. 'Factor 3'), the factor will not be removed ('Keep'). Finally, in the fourth step, CPM normalization and Trimmed Mean of M-values (TMM)-correction is performed using only the samples from the training cohort as eligible samples to calculate the TMM-correction factor. (**B**) Same example for correlation intron-spanning library size as shown in A.2 (left), but here y-axis indicates CPM normalized counts. This graph emphasizes that, for this particular variable, a correlation coefficient up to 1 has to be selected, resulting in selection of genes stable after CPM normalization.

(**C**) Interquartile range distribution of all genes after CPM normalization ordered by correlation with library size. Highly correlated genes (right of black line, example threshold r>0.8) show a minimal interquartile range after CPM normalization as compared to the samples with a diminished correlation coefficient (left of the black line). (**D**) Relative log expression (RLE) plots of 263 samples normalized using our previous approach ((Best et al., 2015), upper plot) and the novel approach (current study, lower plot). The RLE plot indicates the log-ratio of a read count to the median count across samples, and should show for a well-normalized datasets a similar distribution centered around zero. The correction module reduces the intersample variability significantly ($p<0.0001$, $n=263$, two-sided Student's t-test).

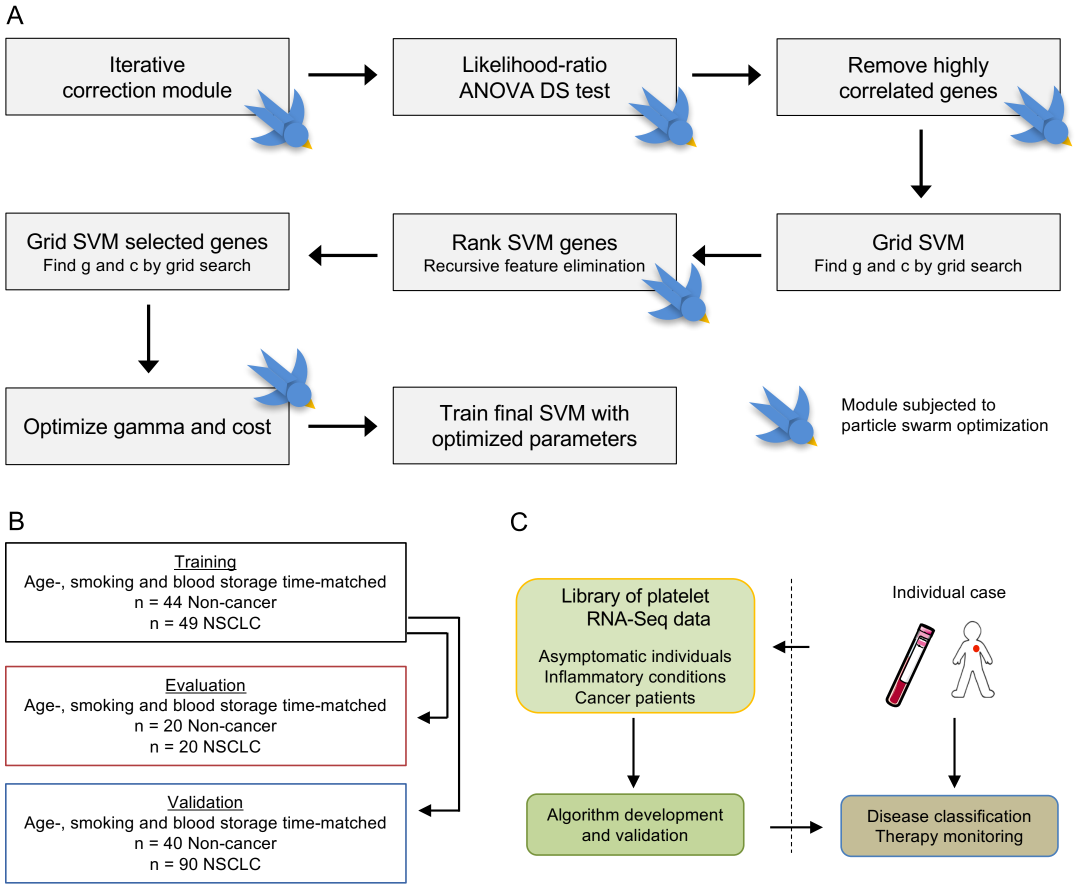

Figure S4, related to Figure 6. Swarm intelligence and thromboSeq.

(**A**) Schematic overview of the PSO-enhanced thromboSeq classification module. Multiple steps and filters of the algorithm are particle swarm-optimized, as indicated by the 'bird'-sign. First, the dataset is subjected to the iterative correction module (see also Figure S3). Second, most differentially spliced (DS) genes are calculated and selected. Third, highly correlated genes among genes selected in the second step are removed. Fourth, a support vector machine (SVM) model is built using the training cohort, optimizing the gamma (g) and cost (c) parameters by a grid search. Fifth, all genes selected for classification are recursively ranked according to the contribution to the SVM model, resulting in a ranked classification gene list. This list is subjected to swarm-based filtering. Sixth, using the reduced gene list an updated SVM model, again with gamma (g) and cost (c) optimization by grid search, is built. Seventh, the gamma (g) and cost (c) values are further optimized by a second particle swarm optimization algorithm. Finally, using the reduced gene list and optimized gamma (g) and cost (c) parameters the final SVM model is built. (**B**) Schematic representation and sample cohort details of the training, evaluation, and validation cohorts. Cohorts are used for

assessing the analytical performance of PSO-enhanced thromboSeq and to investigate the diagnostic classification power in a patient age, smoking and blood storage time-matched cohort. The training cohort included 44 Non-cancer individuals and 49 patients with NSCLC. The algorithm was optimized using a 40-samples evaluation cohort and validated on a 130-samples validation cohort. (**C**) Schematic representation of thromboSeq machine learning-based liquid biopsies for cancer diagnostics. A library of RNA-seq data generated from blood platelets from individuals with different (malignant) diseases and healthy individuals served as input for thromboSeq algorithm development. Following algorithm optimization using the particle swarm optimization-module and model validation, the platform enables RNA signature-based disease classification for individual cases. By nature, swarm intelligence allows for self-reorganization and re-evaluation, enabling continuous algorithm optimization.
